# Mitochondrial transport of catalytic RNAs and targeting of the organellar transcriptome in human cells

**DOI:** 10.1093/jmcb/mjad051

**Published:** 2023-08-17

**Authors:** Paweł Głodowicz, Konrad Kuczyński, Romain Val, André Dietrich, Katarzyna Rolle

**Affiliations:** Department of Molecular Neurooncology, Institute of Bioorganic Chemistry, Polish Academy of Sciences, ul. Z. Noskowskiego 12/14, 61-704 Poznan, Poland; Department of Molecular Neurooncology, Institute of Bioorganic Chemistry, Polish Academy of Sciences, ul. Z. Noskowskiego 12/14, 61-704 Poznan, Poland; Institute of Plant Molecular Biology, French National Center for Scientific Research (CNRS) and University of Strasbourg, 12 rue du Général Zimmer, 67084 Strasbourg, France; Institute of Plant Molecular Biology, French National Center for Scientific Research (CNRS) and University of Strasbourg, 12 rue du Général Zimmer, 67084 Strasbourg, France; Department of Molecular Neurooncology, Institute of Bioorganic Chemistry, Polish Academy of Sciences, ul. Z. Noskowskiego 12/14, 61-704 Poznan, Poland

**Keywords:** human cells, mitochondria, ribozyme, RNA trafficking, transcriptome manipulation, tRNA-like structure

## Abstract

Mutations in the small genome present in mitochondria often result in severe pathologies. Different genetic strategies have been explored, aiming to rescue such mutations. A number of these strategies were based on the capacity of human mitochondria to import RNAs from the cytosol and designed to repress the replication of the mutated genomes or to provide the organelles with wild-type versions of mutant transcripts. However, the mutant RNAs present in mitochondria turned out to be an obstacle to therapy and little attention has been devoted so far to their elimination. Here, we present the development of a strategy to knockdown mitochondrial RNAs in human cells using the transfer RNA-like structure of *Brome mosaic virus* or *Tobacco mosaic virus* as a shuttle to drive *trans*-cleaving ribozymes into the organelles in human cell lines. We obtained a specific knockdown of the targeted mitochondrial *ATP6* mRNA, followed by a deep drop in ATP6 protein and a functional impairment of the oxidative phosphorylation chain. Our strategy provides a powerful approach to eliminate mutant organellar transcripts and to analyse the control and communication of the human organellar genetic system.

## Introduction

The vast majority of mitochondrial proteins are encoded by nuclear genes and imported into the organelles upon cytosolic translation, but the organelles cannot function without the contribution of their own genetic system. The latter provides a set of essential subunits of the oxidative phosphorylation (OXPHOS) complexes located at the mitochondrial inner membrane. The mammalian mitochondrial DNA (mtDNA) is a compact molecule of 16.5 kb, deprived of intergenic regions and introns, that encodes 13 polypeptides, 22 transfer RNAs (tRNAs), and two ribosomal RNAs (rRNAs) and carries a single control region (Saccone et al., 1999). Due to the oxidative environment prevailing in mitochondria, the mtDNA is prone to mutation. Almost 1000 pathogenic base substitutions have been characterized in the human mtDNA, affecting protein, tRNA or rRNA genes, as well as the control region (see http://www.mitomap.org). These include missense mutations in protein-coding genes and point mutations in tRNA or rRNA genes that impair mitochondrial protein synthesis. Genetic dysfunction in the organelles disorganizes the biogenesis of the OXPHOS chain, thereby impairing energetic and metabolic homeostasis of the cells. As a consequence, mtDNA mutations cause severe neurodegenerative diseases (Johnson et al., 2021). For instance, missense mutations can be associated with two common ophthalmologic manifestations, Leber's hereditary optic neuropathy and neuropathy, ataxia, and retinitis pigmentosa, and tRNA point mutations are associated with the myoclonic epilepsy with ragged-red fibers (MERRF) syndrome and the mitochondrial encephalopathy with lactic acidosis and stroke-like episodes (MELAS) syndrome. Mutations can affect all mtDNA copies in the cell (homoplasmic state) or only some copies (heteroplasmic state).

A wide range of strategies have been developed for treating mitochondrial genetic diseases (Hirano et al., 2018; Russell et al., 2020), including manipulation of the content or turnover of mitochondria in cells and mitochondrial genome engineering (reviewed in Silva-Pinheiro and Minczuk, 2022). Editing of the mtDNA through the CRISPR/Cas technology has been evaluated but still lacks robust evidence of success (reviewed in Yin et al., 2022). Double-stranded DNA deaminase-derived cytosine base editors have recently emerged as a tool to introduce desired edits into the mtDNA (Mok et al., 2022; Guo et al., 2022). Further approaches to complementing mtDNA mutations include allotopic expression and mitochondrial targeting sequence-directed organellar import of functional versions of mitochondrial proteins otherwise encoded by mutated mtDNA genes (Kaltimbacher et al., 2006; Bonnet et al., 2007, 2008; Ellouze et al., 2008). As many disorders of the mitochondrial genetic system are caused by mutations in organellar tRNA genes, there is also special interest in rescuing these mutations by importing normal copies of the relevant tRNA from the cytosol. Indeed, in many organisms, mitochondria are able to take up various types of RNAs (Salinas et al., 2008; Sieber et al., 2011; Wang et al., 2012a; Warren and Sloan, 2020). In particular, when the set of tRNAs encoded by the mtDNA is not sufficient to support protein synthesis, mitochondria import some of the nuclear-encoded tRNAs from the cytosol. Although possessing an apparently complete set of tRNA genes, human mitochondria were proved able to import yeast or human tRNA^Lys^ variants *in vitro* (Entelis et al., 2001). Mitochondrial import of tRNA^Gln^ was also implied in human cells (Rubio et al., 2008). Yeast tRNA^Lys^ variants expressed from nuclear transgenes in human cell lines carrying the tRNA^Lys^ 8344A>G MERRF mutation were imported into mitochondria to rescue the mitochondrial functional deficiency (Kolesnikova et al., 2004). Similarly, nuclear expression of recombinant tRNAs with a leucine identity in human cell lines allowed a significant rescue of the mitochondrial tRNA^Leu^ 3243A>G mutation, the major cause of the MELAS syndrome (Karicheva et al., 2011).

Building on the natural process of tRNA import from the cytosol into mitochondria in plant cells (Salinas et al., 2008; Warren and Sloan, 2020), we previously established an aminoacylatable tRNA mimic that can be used *in vivo* as a shuttle for importing cargo RNAs expressed from nuclear transgenes into plant mitochondria (Val et al., 2011). Since the set of mitochondrially importable tRNAs in plants includes tRNA^Val^ isoacceptors (Salinas et al., 2008), we chose the valine-specific tRNA-like structure (TLS) naturally present at the 3′-end of the *Turnip yellow mosaic virus* (TYMV) genomic RNA as a mitochondrial shuttle (Matsuda and Dreher, 2004). When *trans*-cleaving hammerhead ribozymes as cargo sequences were attached as 5′-end trailors to TYMV TLS (termed PKTLS), specific knockdown of mitochondrial RNAs was obtained (Val et al., 2011; Sultan et al., 2016; Niazi et al., 2019). In the present work, we first used TYMV PKTLS (the last 120 nucleotides at the 3'-end of the viral genomic RNA) to shuttle custom sequences into mitochondria in human cells, but the attempts were not successful (details in Supplementary material). Thus, we switched to two other plant viral TLSs from *Brome mosaic virus* (BMV) and *Tobacco mosaic virus* (TMV) (Mans et al., 1991; Gultyaev et al., 1994), which have tyrosine (BMV) and histidine (TMV) aminoacylation specificity, respectively (Mans et al., 1991), and are both proved able to drive *trans*-cleaving hammerhead ribozymes attached as 5′-end trailors into the organelles. We demonstrated that BMV TLS and TMV TLS were highly enriched in mitochondria upon the induced nuclear expression in HEK293 and HepG2 human cell lines. The imported catalytic RNAs turned out to be active in mitochondria, which led to specific knockdown of their target RNA in the organelles and the corresponding protein. We have thus developed a novel approach to manipulating the mitochondrial transcriptome in human cells, contributing to mitochondrial gene therapy strategies.

## Results

### BMV TLS and TMV TLS are targeted to mitochondria in human cells

**Figure 1:**
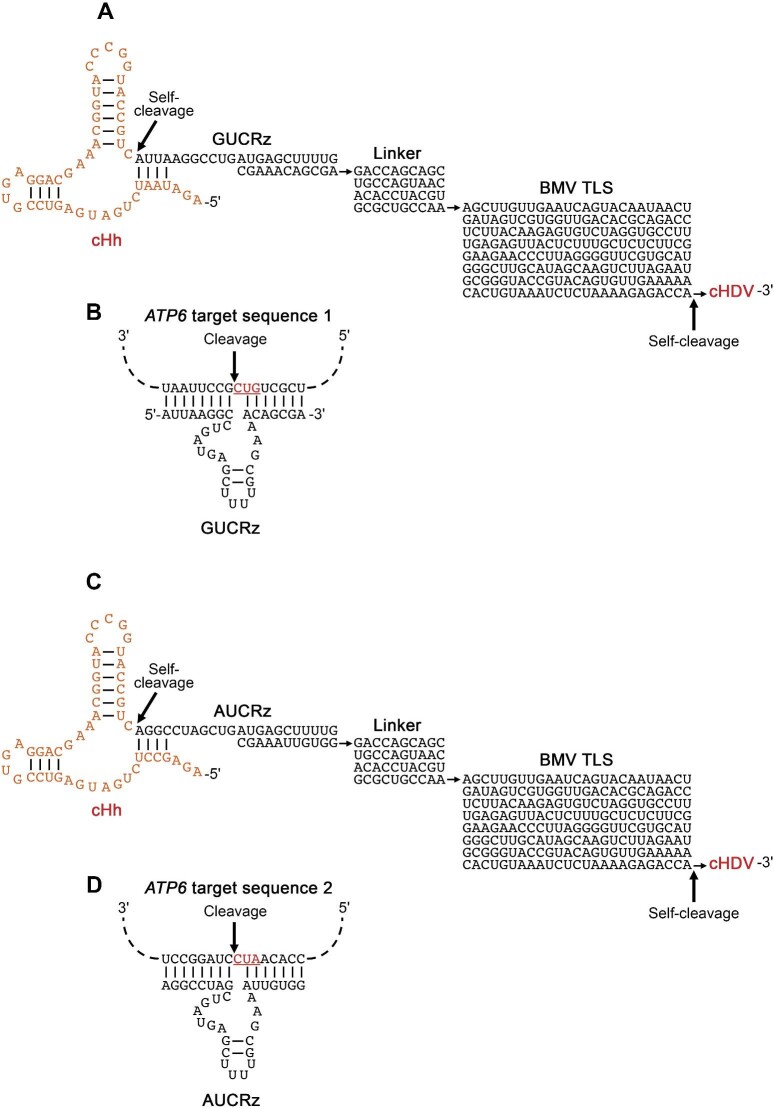
Schematic representation of the primary transcript deriving from the transgene in human cells stably transformed with the pcDNA5-cHh-GUCRz-L-BMVTLS-cHDV or pcDNA5-cHh-AUCRz-L-BMVTLS-cHDV construct. (**A** and **C**) Following self-cleaving of the 5′ cHh ribozyme and the 3′ cHDV ribozyme, the final product contains the GUCRz *trans*-ribozyme (**A**) or AUCRz *trans*-ribozyme (**C**) sequence combined with BMV TLS through the linker. (**B** and **D**) Schematic representation of the GUCRz *trans*-ribozyme (**B**) or the AUCRz *trans*-ribozyme (**D**) annealed to the corresponding target sequence in mitochondrial *ATP6* mRNA.

To establish whether BMV TLS and/or TMV TLS can be translocated into mitochondria in human cells and possibly drive a cargo sequence into the matrix, various expression constructs were built. The BMV TLS (Figure 1A and C) and TMV TLS (Supplementary Figure S4) sequences, i.e. in each case the last 200 nucleotides (nt) from the 3′-end of the genomic RNA, were assembled with the sequence encoding a *trans*-cleaving hammerhead ribozyme against mitochondrial *ATP6* mRNA, so that target cleavage would be the ultimate proof for full import of the chimeric RNA into the organellar matrix.

Two target sites were selected in the *ATP6* sequence to design *trans*-cleaving ribozymes, based on either a GUC (Figure 1B) or an AUC (Figure 1D) triplet. The corresponding catalytic RNAs, called GUCRz and AUCRz, were designed as previously described (Val et al., 2011; Sultan et al., 2016; Niazi et al., 2019). The TLS and the *trans*-ribozyme in each case were separated by a weakly structured linker sequence (L) (Supplementary Materials and  methods). The four genetic constructs (pcDNA5-cHh-GUCRz-L-BMVTLS-cHDV, pcDNA5-cHh-AUCRz-L-BMVTLS-cHDV, pcDNA5-cHh-GUCRz-L-TMVTLS-cHDV, and pcDNA5-cHh-AUCRz-L-TMVTLS-cHDV, respectively) were used to transform both commercial Flp-In T-REx 293 cells (deriving from HEK293 human kidney cells) and home-made Flp-In HepG2 cells (carrying the pFRT/lacZeo plasmid and the pcDNA6/TR plasmid; Supplementary Materials and methods). The stable transformants generated were quoted as ‘HEK’ or ‘Hep’ transformants in the following study.

Then, the induction of transgene expression in these transformants was tested with increasing tetracycline concentrations. Real-time quantitative polymerase chain reaction (RT-qPCR) was performed to determine transgene expression levels in samples collected on Day 1 after onset of tetracycline treatment (Supplementary Figures S5A, B and S6A, B). Counting alive cells within the cultures through the MTT test (Mosmann, 1983) showed no significant cytotoxicity of tetracycline up to 10 µg/ml (Supplementary Figure S7). This concentration was thus used for all subsequent experiments.

**Figure 2:**
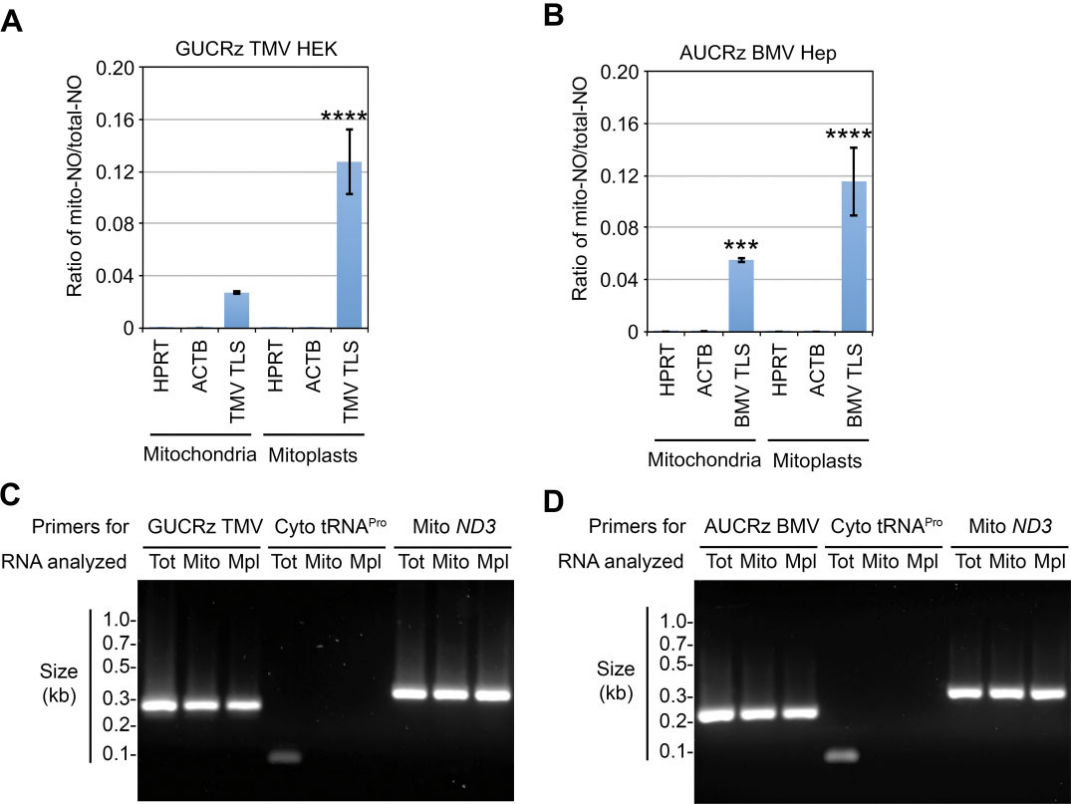
The GUCRz-L-TMV TLS or AUCRz-L-BMV TLS chimeric RNA is targeted to mitochondria in HEK or Hep cells. RNAs were prepared from whole-cell lysates, intact mitochondria , and mitoplasts of HEK cells stably transformed with the pcDNA5-cHh-GUCRz-L-TMVTLS-cHDV construct (**A** and **C**, hereafter referred to as GUCRz TMV HEK transformants) or Hep cells stably transformed with the pcDNA5-cHh-AUCRz-L-BMVTLS-cHDV construct (**B** and **D**, hereafter referred to as AUCRz BMV Hep transformants) harvested on Day 1 after transgene induction with 10 µg/ml tetracycline. (**A** and **B**) RT-qPCR for the nuclear transcripts hypoxanthine phosphoribosyl transferase (HPRT) and actine B (ACTB), TMV TLS, and BMV TLS. The results are presented as the mitochondrial-NO/total-NO ratio. Data from three independent biological replicates were analysed with the Student's *t*-test; ***P < 0.001; ****P < 0.0001. Data represent mean ± SD. (**C** and **D**) Regular PCR analysis of total (Tot), mitochondrial (Mito), and mitoplast (Mpl) RNA samples for expression of the GUCRz TMV transcript, the AUCRz BMV transcript, the cytosol-specific tRNA^Pro^(AGG3-1) (Cyto tRNA^Pro^), and mitochondrial *ND3* mRNA (Mito *ND3*). PCR products were fractionated on agarose gel.

In order to establish whether BMV TLS and TMV TLS were targeted to the organelles in intact human cells, mitochondria were isolated from transformed HEK and Hep cell lines on Day 1 after transgene induction. An enrichment of the *trans*-ribozyme-L-TLS chimeric transcripts in mitochondrial fractions was established by RT-qPCR probing (Figure 2A and B). The mitochondria were subjected to hypotonic shock to generate mitoplasts and treated with RNase prior to RNA extraction. RT-qPCR probing showed additional enrichment of the *trans*-ribozyme-L-TLS chimeric transcripts in mitoplast fractions (Figure 2A and B). Then, the total, mitochondrial, and mitoplast RNAs were reverse-transcribed followed by PCR with forward primers from the ribozyme moiety of the GUCRz-L-TMV TLS or AUCRz-L-BMV TLS sequence and reverse primers from the 3′ region of TMV TLS or BMV TLS (Supplementary Table S1), with the cytosol-specific tRNA^Pro^(AGG3-1) as a contamination control and the *ND3* mRNA as a representative of mitochondrial transcripts. As shown in Figure 2C and D, the anti-*ATP6 trans*-ribozyme-L-TMV TLS and anti-*ATP6 trans*-ribozyme-L-BMV TLS transcripts are detected in both mitochondrial and mitoplast fractions, without significant cytosolic contamination. The identical sizes of the obtained PCR products confirmed that the complete ribozyme-L-TLS transcripts could be recovered in mitochondria. The levels of ribozyme-L-TLS transcripts in the organelles of transformant cell lines also appeared to be similar to that of endogenous mitochondrial transcripts such as *ND3*. These data altogether implied that our chimeric RNAs were translocated into the mitochondrial matrix *in vivo*.

### Mitochondrial translocation of a catalytic RNA driven by BMV TLS or TMV TLS leads to specific organellar target RNA cleavage and knockdown

**Figure 3:**
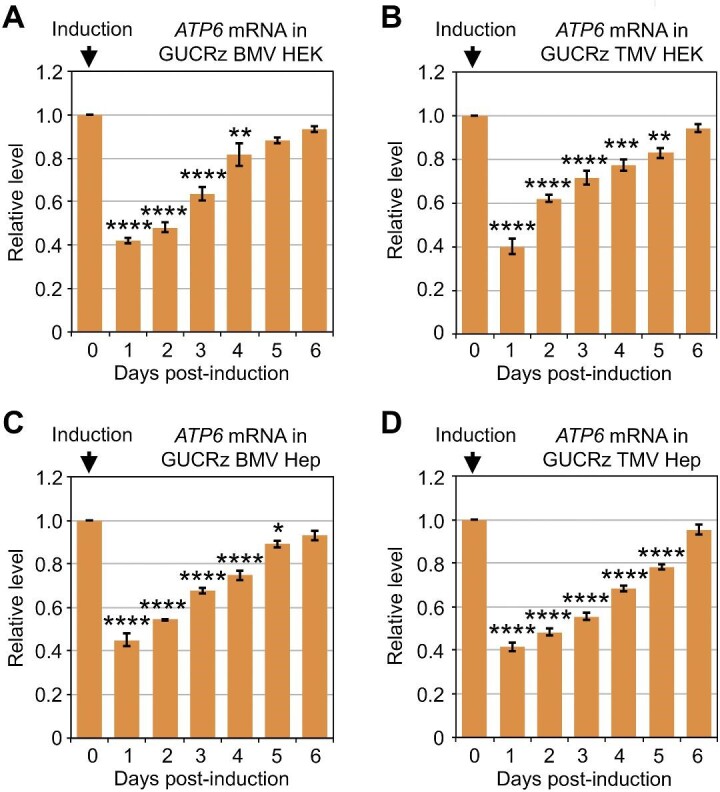
Expression of the GUCRz-L-BMV TLS or GUCRz-L-TMV TLS chimeric RNA in HEK or Hep transformants results in mitochondrial *ATP6* mRNA knockdown. Transgene expression was induced by 10 µg/ml tetracycline in cells stably transformed with the pcDNA5-cHh-GUCRz-L-BMVTLS-cHDV construct (**A** and **C**, referred to as GUCRz BMV HEK or Hep transformants) or pcDNA5-cHh-GUCRz-L-TMVTLS-cHDV construct (**B** and **D**, referred to as GUCRz TMV HEK or Hep transformants), and cells were harvested every day until Day 6. Total RNA was extracted, and steady-state levels of the mitochondrial *ATP6* mRNA were analysed by RT-qPCR. Data from three independent biological replicates were analysed with the Student's *t*-test; *P < 0.05; **P < 0.01; ***P < 0.001; ****P < 0.0001. Data represent mean ± SD.

Whether BMV TLS and TMV TLS can be translocated into mitochondria and drive a trailor sequence into the matrix was further examined by assessing the activity of the *trans*-ribozyme against mitochondrial *ATP6* mRNA. Cells were harvested every day until Day 6, and the relative levels of various mitochondrial mRNAs along with the expression of the constructs were determined by RT-qPCR. Expression of the anti-*ATP6 trans*-ribozymes combined with BMV TLS or TMV TLS in HEK or Hep cells led to 60% drop in the steady-state level of mitochondrial *ATP6* mRNA on Day 1 post-induction (Figure 3; Supplementary  Figure S8), when the transgene expression was at the highest level (Supplementary Figures S5C, D and S6C, D). The knockdown was then progressively released along with the progressive decrease in the level of anti-*ATP6 trans*-ribozyme-TLS transcript. In all cases, *ATP6* steady-state level was recovered to the initial level until Day 6 post-induction, when the level of anti-*ATP6 trans*-ribozyme-TLS transcript was negligible. Meanwhile, the steady-state level of mitochondrial *COX1, COX2, ND2*, or *ND5* mRNA was unaffected (Supplementary Figure S9), excluding an overall detrimental effect on mitochondria and confirming the specific *trans*-ribozyme-mediated knockdown of the *ATP6* mRNA in the organelles upon BMV TLS- or TMV TLS-driven translocation of the trailor sequence.

**Figure 4:**
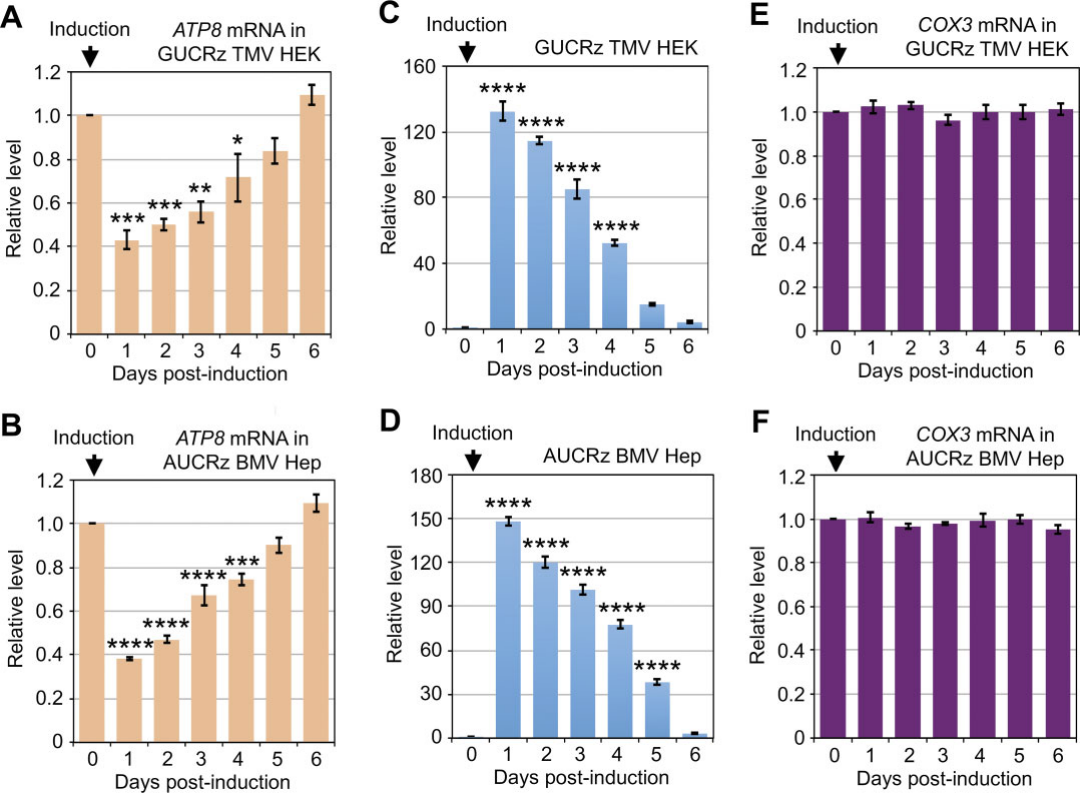
Expression of the GUCRz-L-TMV TLS chimeric RNA in HEK transformants or the AUCRz-L-BMV TLS chimeric RNA in Hep transformants affects *ATP8* but not *COX3* mRNA levels. Transgene expression was induced by 10 µg/ml tetracycline in GUCRz TMV HEK transformants (**A, C**, and **E**) or AUCRz BMV Hep transformants (**B, D**, and **F**), and cells were harvested every day until Day 6. Total RNA was extracted, and transgene expression (**C** and **D**) and steady-state levels of the *ATP8* (**A** and **B**) and *COX3* (**E** and **F**) mRNAs were analysed by RT-qPCR. Data from three independent biological replicates were analysed with the Student's *t*-test; *P < 0.05; **P < 0.01; ***P < 0.001; ****P < 0.0001. Data represent mean ± SD.

**Figure 5:**
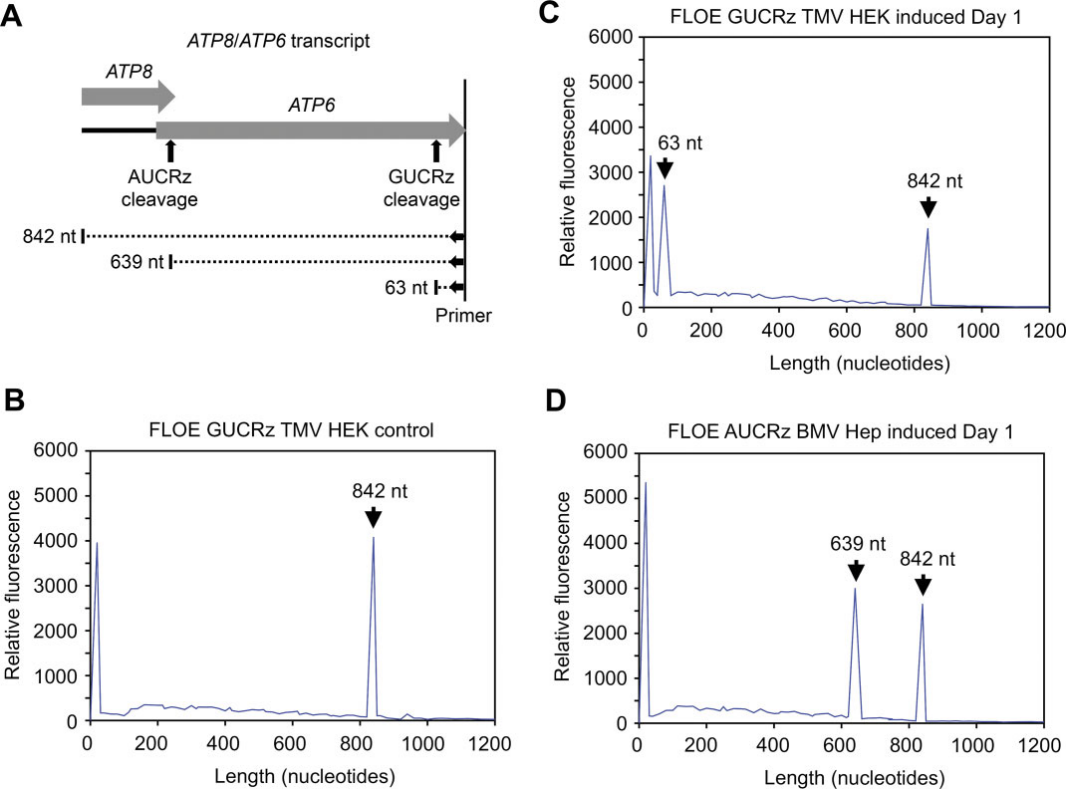
*In viv*o cleavage of mitochondrial *ATP6* mRNA. (**A**) Scheme illustrating the FLOE products expected from full-length *ATP8*/*ATP6* transcript (842 nt) and 3′ fragments resulting from cleavage by the AUCRz ribozyme (639 nt) and the GUCRz ribozyme (63 nt), using the atp6_atp8_FAM primer (Supplementary Table S1). (**B**–**D**) FLOE analysis of total RNA extracted from control uninduced GUCRz TMV HEK transformants (**B**) or 10 µg/ml tetracycline-treated GUCRz TMV HEK transformants (**C**) or AUCRz BMV Hep transformants (**D**) harvested on Day 1 post-induction. The profiles for Day 2 and Day 3 post-induction are shown in Supplementary Figure S11. The expected products are indicated by arrows. The peak present in all profiles on the extreme left corresponds to the left over of primer.

The *ATP8* gene is located upstream of the *ATP6* gene in the mtDNA and its 3′-end overlaps by 46 nt with the 5′-end of *ATP6*, while the start codon of the *COX3* gene located downstream overlaps with the stop codon of *ATP6. ATP8, ATP6*, and *COX3* are initially expressed in one polycistronic transcript that is subsequently processed into *ATP8*/*ATP6* and *COX3* mRNAs (Dautant et al., 2018; Jedynak-Slyvka et al., 2021). The *ATP8*/*ATP6* bicistronic transcript is translated through a single ribosome/mRNA engagement, while *COX3* is translated separately (Cruz-Zaragoza et al., 2021). In line with this scheme, the steady-state level of *ATP8* exhibited the same dynamic pattern as that of *ATP6* upon expression of the anti-*ATP6 trans*-ribozymes driven by BMV TLS or TMV TLS in HEK or Hep cells (Figure 4A and B), while the steady-state level of *COX3* was unaffected (Figure 4E and F), along with the decrease in the level of anti-*ATP6 trans*-ribozyme-TLS transcript (Figure 4C and D). It is worth mentioning that the same effect on the *ATP8* mRNA level was observed by expressing the AUCRz anti-*ATP6* ribozyme, which targets a sequence in the *ATP8*/*ATP6* overlap, or the GUCRz anti-*ATP6* ribozyme, which targets a sequence in the 3′-end of *ATP6* (Figure 5A).

Cells under hypoxic conditions switch from OXPHOS to glycolysis (Kierans and Taylor, 2021). The resulting reduction in oxidative metabolism downregulates the expression of mitochondrial OXPHOS genes (Arnaiz et al., 2021), including *ATP6*. Expression of the anti-*ATP6 trans*-ribozyme-TLS chimeric RNAs in HEK or Hep cells cultivated under hypoxic conditions led to further knockdown of the *ATP6/ATP8* transcript. Both *ATP6* and *ATP8* mRNA levels on Day 3 post-induction were <20% of the levels on Day 0, while ∼50% of mRNA levels on Day 0 were kept in uninduced cells not expressing an anti-*ATP6 trans*-ribozyme (Supplementary Figure S10).

Fluorescently labeled oligonucleotide extension (FLOE) assays with a specific 3′ primer detected a single *ATP8*/*ATP6* transcript of 842 nt in mitochondrial RNA samples from control uninduced transformants (Figure 5A and B; Supplementary Figure S11B). The level of this 842-nt product was reduced in mitochondrial RNA samples from tetracycline-treated transformants harvested on Day 1 post-induction, accompanied with a new signal corresponding to the 63-nt or 639-nt 3′ fragment expected from cleavage of the *ATP8*/*ATP6* transcript by the GUCRz or AUCRz ribozyme, respectively (Figure 5A, C, and D). Both levels of the 63-nt and 639-nt products subsequently decreased on Day 2 and Day 3 post-induction, along with a progressive re-increase of the 842-nt full-length *ATP8/ATP6* transcript level (Supplementary Figure S11) and the decreasing ribozyme level (Figure 4C and D).

### Specific organellar RNA knockdown leads to functional impairment of the OXPHOS chain in mitochondria

**Figure 6:**
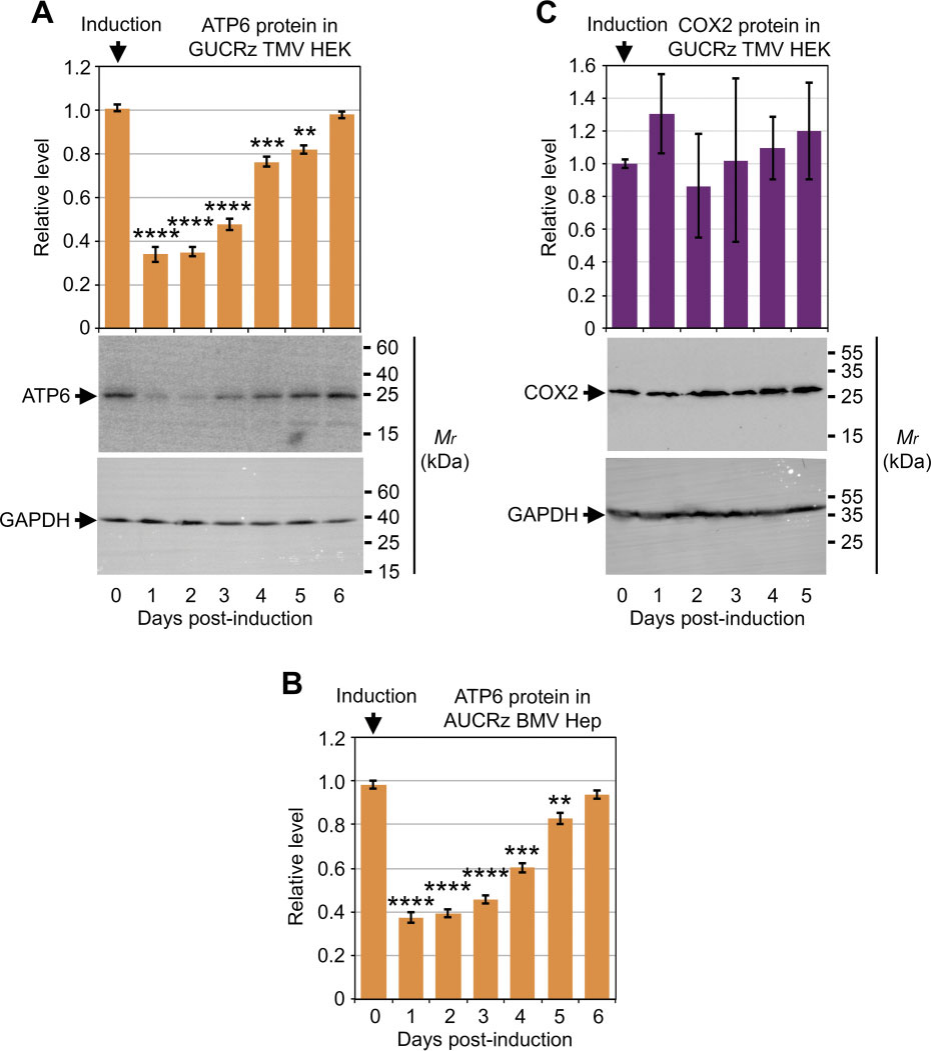
The ATP6 protein level decreases upon *trans*-ribozyme-mediated knockdown of mitochondrial *ATP6* mRNA. Transgene expression was induced by 10 µg/ml tetracycline in GUCRz TMV HEK transformants (**A** and **C**) and AUCRz BMV Hep transformants (**B**), and cells were harvested every day until Day 6. Total protein was extracted and analysed by western blotting with anti-ATP6, anti-COX2, and anti-GAPDH antibodies. Representative western blots are shown. Steady-state levels of ATP6 and COX2 were normalized against GAPDH levels. Data from three independent biological replicates were analysed with the Student's *t*-test; **P < 0.01; ***P < 0.001; ****P < 0.0001. Data represent mean ± SD.

To determine the consequences of the ribozyme-mediated *ATP6* mRNA knockdown in mitochondria, protein samples were extracted from HEK transformants expressing the GUCRz-L-TMV TLS transcript and Hep transformants expressing the AUCRz-L-BMV TLS transcript every day until Day 6 post-induction and subjected to western blot analysis with antibodies against the mitochondrial ATP6 polypeptide. The ATP6 protein level decreased by >60% on Day 1 and Day 2 post-induction (Figure 6A and B), reflecting a sufficient knockdown of the *ATP6* mRNA (Figure 3B; Supplementary Figure S8C). Subsequently, the ATP6 protein level progressively re-increased along with the progressive release of the *ATP6* mRNA knockdown and completely recovered on Day 6 post-induction (Figures 3B and 6A, B; Supplementary Figure S8C). Reduction of the ATP6 protein level was thus in line with TMV TLS- or BMV TLS-mediated organellar import of a functional GUCRz or AUCRz ribozyme and specific knockdown of the *ATP6* mRNA in mitochondria. Remarkably, no delay was observed in protein knockdown following mRNA knockdown, implying continuous translation limited by mRNA availability and rapid turnover of the ATP6 protein. Furthermore, there was no drop in the level of other mitochondrial proteins such as COX2 upon expression of the anti-*ATP6* ribozyme-TLS chimeric RNAs, confirming the specificity of these results (Figure 6C).

**Figure 7:**
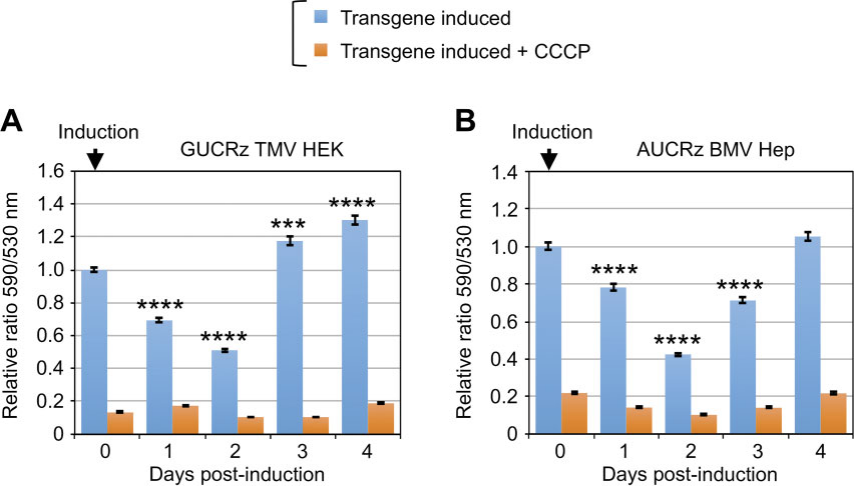
The mitochondrial membrane potential is impaired upon *trans*-ribozyme-mediated knockdown of mitochondrial *ATP6* mRNA and ATP6 protein. Transgene expression was induced by 10 µg/ml tetracycline in GUCRz TMV HEK transformants (**A**) and AUCRz BMV Hep transformants (**B**), and cells were harvested every day until Day 4. The cells were incubated for 30 min with JC-1 dye, and the fluorescence was measured. The results are presented as the ratio of the fluorescence at 590 nm (JC-1 dye translocated into the organelles) to the fluorescence at 530 nm (JC-1 dye remaining in the cytosol), reflecting the mitochondrial membrane potential status in cells (blue bars). Addition of the uncoupler CCCP was used as a control showing full dissipation of the transmembrane chemical proton gradient (ΔpH) and electric potential (ΔΨ) (orange bars). Data from three independent biological replicates were analysed with the Student's *t*-test; ***P < 0.001; ****P < 0.0001. Data represent mean ± SD.

We further examined whether *ATP6* mRNA knockdown and ATP6 protein knockdown in mitochondria have a functional impact on the OXPHOS chain, especially the inner membrane complex that contains ATP6. First, we analysed the mitochondrial membrane potential in the transformants by measuring membrane potential-driven mitochondrial uptake of the fluorescent dye. Expression of the anti-*ATP6 trans*-ribozymes combined with BMV TLS or TMV TLS led to a drop in organellar dye uptake starting on Day 1 post-induction and achieving 50%–60% reduction on Day 2 (Figure 7A and B; Supplementary Figures S12 and S13). The drop in dye uptake was then progressively released along with the progressive decrease in the level of anti-*ATP6 trans*-ribozyme-TLS transcript. Thus, in line with the knockdown of the *ATP6* mRNA and the ATP6 protein, expression of the anti-*ATP6 trans*-ribozyme-TLS chimeric RNAs affected the mitochondrial OXPHOS function, strongly reducing the transient membrane potential (Supplementary Figures S12 and S13).

**Figure 8:**
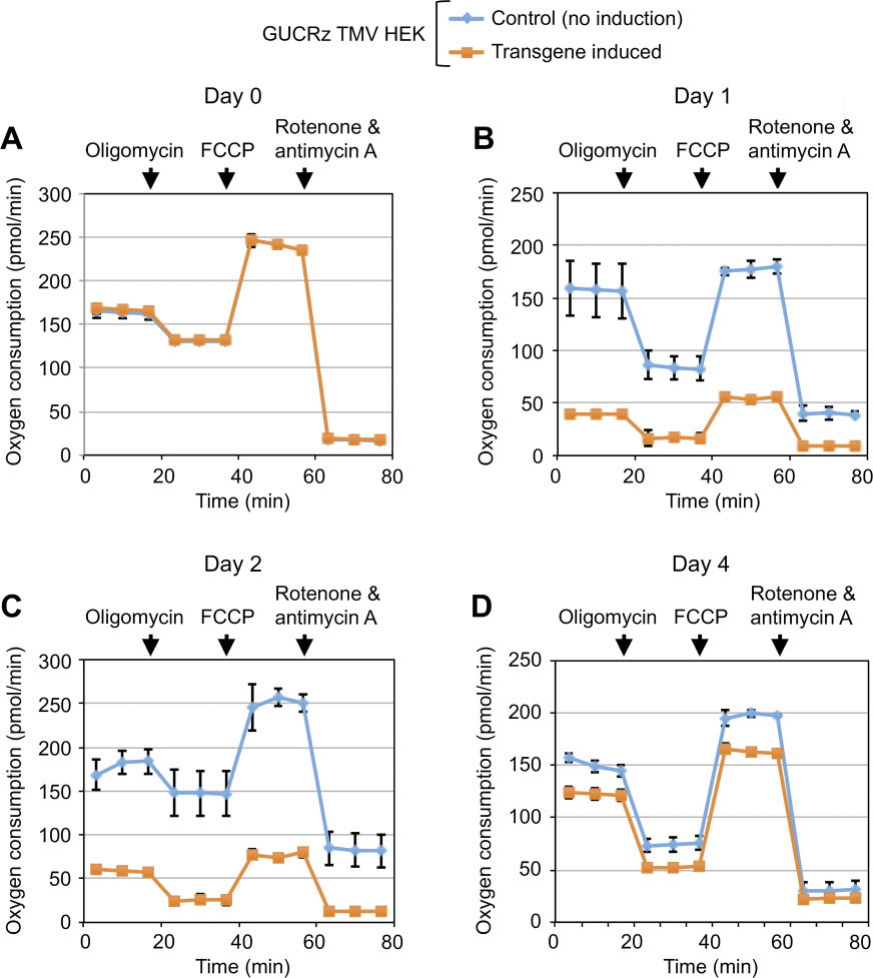
The activity of the mitochondrial OXPHOS chain is impaired upon *trans*-ribozyme-mediated knockdown of mitochondrial *ATP6* mRNA and ATP6 protein. Control uninduced (organe) or 10 µg/ml tetracycline-treated (transgene-induced, blue) GUCRz TMV HEK transformants were cultured in Agilent Seahorse microplates. Oxygen consumption in cells was measured every day until Day 4 in an Agilent Seahorse analyser, with addition of different effectors as indicated (oligomycin, FCCP, and rotenone and antimycin A). Data from three independent biological replicates were analysed and presented as mean ± SD.

Then, we analysed the activity of the OXPHOS chain in the transformants by measuring cellular oxygen consumption every day until Day 4. As shown in Figure 8, basal oxygen consumption, ATP synthase-linked oxygen consumption, and maximal oxygen consumption in HEK cells expressing the GUCRz-L-TMV TLS chimeric RNA were identical to those in control uninduced cells on Day 0, remarkably decreased on Day 1 and Day 2, re-increased from Day 3, and again close to those in control cells on Day 4, which was in agreement with the induced expression and consequently progressive decrease in the level of anti-*ATP6 trans*-ribozyme-TLS transcript. Similar oxygen consumption patterns were observed in Hep cells expressing the AUCRz-L-BMV TLS chimeric RNA (Supplementary Figure S14).

Altogether, our results demonstrate that expression and mitochondrial translocation of the anti-*ATP6 trans*-ribozyme-TLS transcripts could lead to a cascade of effects from knockdown of the *ATP6* mRNA to reduction of the ATP6 protein level and impairment of the OXPHOS chain activity. The decreased ATP6 level restricts proton exchange through the F0 domain of ATP synthase, which negatively affects respiration and impairs the organellar membrane potential.

## Discussion

Over the years, the mechanism of mitochondrial RNA trafficking in human cells has evolved considerably, from the initial idea of a completely autonomous organellar genetic system to the currently emerging picture of an extensive and diverse exchange with the cytosol (Weber-Lotfi and Dietrich, 2018; Huang et al., 2021). Contrary to the findings in many other organisms (Salinas-Giegé et al., 2015), the set of 22 mtDNA-encoded tRNAs originally seemed sufficient to support mitochondrial translation in human organelles due to the non-canonical structure of some isoacceptors and adaptations of the genetic code (Suzuki et al., 2011). Thus, whether a tRNA can be translocated into human mitochondria is not predictable and needs to be examined on a case-by-case basis. Further studies have demonstrated mitochondrial import of tRNA^Gln^ isoacceptors from the cytosol (Rubio et al., 2008) and *in vitro* uptake of yeast tRNA^Lys^ CUU derivatives (Entelis et al., 2001) and bacterial pre-tRNA^Asp^ (Rubio et al., 2008). In addition, import of a functional yeast tRNA^Lys^ into mitochondria in human cells carrying the mtDNA A8344G pathogenic tRNA^Lys^ mutation, which can cause the MERRF syndrome, led to a partial rescue of the mitochondrial functions affected by the mutation (Kolesnikova et al., 2004). Similarly, expression of recombinant tRNAs bearing the identity elements for human mitochondrial leucyl-tRNA synthetase could partially rescue the MELAS syndrome caused by the A3243G pathogenic mutation in the mtDNA tRNA^Leu^ (UUR) gene (Karicheva et al., 2011).

Our results imply that tRNAs^His^ and tRNAs^Tyr^, rather than tRNAs^Met^ and tRNAs^Val^, are more likely to be importable into human organelles. The sorting results of tRNA^His^ (TMV TLS) and tRNA^Tyr^ (BMV TLS) identities vs. tRNA^Val^ (TYMV TLS) and tRNA^Met^ (mutated TYMV TLS) identities suggest that our ribozyme-TLS transcripts follow a mitochondrial import mechanism involving the recognition by cognate human aminoacyl-tRNA synthetases. Self-cleavage of the *cis*-HDV ribozyme in our transcripts unmasks the TLS 3′-CCA end. However, whether such aminoacylation occurs in human cells and plays a role in mitochondrial translocation of BMV TLS or TMV TLS remains an open question. Notably, in infected plant cells, the full TYMV genomic RNA is functionally charged *in vivo* and eEF1a binding to the aminoacylated viral RNA represses minus strand synthesis (Matsuda et al., 2004). Since RT-qPCR analysis could not establish the size of the transgene-derived chimeric RNA, which depends on the cleavage efficiency of the 5′-*cis*-hammerhead ribozyme and the 3′-*cis*-HDV ribozyme, the persistence of processing intermediates can thus not be excluded. As *cis*-HDV cleavage determines whether or not the 3′-end is the chargeable CCA, complementary analyses, for instance FLOE assays with different primers, could confirm that the RNAs imported into mitochondria correspond to the expected 5′-*trans*-ribozyme-L-TLS-3′ transcripts.

Our valine-specific TYMV TLS shuttle that were able to drive *trans*-cleaving ribozymes into mitochondria in plants and manipulate the organellar transcriptome (Val et al., 2011; Sultan et al., 2016; Niazi et al., 2019) appears eligible for the translocation to the inter-membrane space through the outer membrane of human mitochondria but cannot reach the matrix subcompartment. This suggests a two-step discrimination of importable RNAs, taking place successively at the outer membrane and inner membrane, possibly leading to a differential process. Such a concept might be of general relevance. It has been proposed that the organelles might constitute a ‘reservoir’ or a ‘warehouse’ for storage and ‘on demand’ release of miRNAs for regulatory processes or stress response (Bandiera et al., 2013; Wang and Springer, 2015). Some reports were based on analyses of RNase-treated organelles with an intact outer membrane, leaving the possibility that, like TYMV TLS, a set of miRNAs or other ncRNAs could be stopped in the mitochondrial intermembrane space after lower stringency translocation through the outer membrane. This is the case for the cytosolic 28S rRNA, which is considered to be mitochondrially imported but degraded in the intermembrane space (Huang et al., 2018). It might also apply to the large human cytomegalovirus Beta2.7 lncRNA, which targets the NDUFA13 (Grim-19) protein of complex I in the electron transport chain (Reeves et al., 2007). Indeed, the NDUFA13 subunit is readily accessible in intermembrane space (Hua et al., 2017). Remarkably, Gao et al. (2021) robustly showed that siRNAs transfected into mouse or human cells are indeed translocated into the mitochondrial matrix. The transfected siRNAs targeting known Ago2-binding sites functioned effectively at the RNA level in mitochondria, leading to knockdown of the corresponding organellar target transcripts. Similar effects were observed when siRNAs were derived from plasmid-expressed shRNAs. These results indicate the occurrence of RNA interference depending on Ago2 slicing activity in mammalian organelles (Gao et al., 2021).

Previous studies from other groups also built on derivatives or signal sequences of RNAs considered as importable to design mitochondrial shuttle RNAs. Short importable RNAs were derived from the *Saccharomyces cerevisiae* tRNA^Lys^ CUU with the D-arm linked by a central domain to the so-called F-hairpin (Kolesnikova et al., 2010). Using a short antigenomic sequence as a central linker could repress the replication of mutated mtDNA in human cells (Comte et al., 2013; Tonin et al., 2014). Mitochondrially importable recombinant versions of the 5S rRNA were prepared by exchanging part of the β domain for short foreign cargo sequences of 12–14 nt (Smirnov et al., 2008; Comte et al., 2013).

Mitochondrial import of the *MRP* RNA and the *H1* RNA (originally described as the RNA component of RNase P) in human cells has been a controversial issue for a long time (Wang et al., 2010; Cannon et al., 2015; Noh et al., 2016; reviewed in Weber-Lotfi and Dietrich, 2018). Nevertheless, the predicted 20-nt stem-loop structures in both RNAs were determined to be mitochondrial import signals. Upon addition of the *MRP* or *H1* RNA stem-loop, the *GAPDH* mRNA was taken up into isolated yeast mitochondria (Wang et al., 2010, 2012b). Moreover, human mitochondrial tRNA^Trp^ fused with the *H1* RNA stem-loop could be imported into isolated mouse liver mitochondria (Wang et al., 2010). The same stem-loop could promote uptake of mitochondrial tRNA precursors into isolated mouse or human cybrid mitochondria and enable translocation of the *COX2* mRNA into mitochondria in human or mouse cells (Wang et al., 2012b). Moreover, when wild-type mitochondrial tRNA precursors were combined with both the *H1* RNA stem-loop and the 3′-UTR sequence that normally targets the mRNA of human mitochondrial ribosomal protein S12 to the organellar surface for translation, the expressed chimeric RNAs could rescue mtDNA mutations affecting tRNA genes in human cybrid cell lines (Wang et al., 2012b).

In addition to repressing mutated mtDNA replication and replacing mutated mitochondrial RNAs, organellar antisense strategies were also tested. Identified as a determinant for mitochondrial import (Bhattacharyya et al., 2002), the D-arm hairpin of the *Leishmania* tRNA^Tyr^ was fused with short RNAs antisense to mitochondrial mRNAs, antisense DNA oligonucleotides, or long polycistronic RNAs carrying native mtDNA coding sequences. The fusions were loaded on an import complex isolated from *Leishmania tropica* or the so-called MITO-Porter nanovesicles and then either incubated with human HepG2 cells or patient-derived cybrids or injected into rats. All expected mitochondrial antisense effects, i.e. mtDNA mutation rescue, stimulation of mitochondrial translation and respiratory capacity, and target RNA knockdown, were observed (Mahato et al., 2011; Jash et al., 2012; Furukawa et al., 2015).

A specific isoform of the 5S rRNA, as well as the *MRP* or *H1* RNA stem-loop, combined with antisense sequences against the translation start site of mitochondrial *ATP6* or *COX2* mRNA reduced levels of the corresponding proteins in the organelles (Towheed et al., 2014). A similar strategy was subsequently developed to target the wild-type *ATP6* sense mRNA into mitochondria in a fly model of mitochondrial encephalomyopathy (ME) resulting from mtDNA mutation in the *ATP6* gene (Markantone et al., 2018). Remarkably, ME rescue only occurred when organellar targeting of the wild-type *ATP6* mRNA was combined with targeting of an antisense sequence against the native *ATP6* translation start site to inhibit the expression of the mutated ATP6 protein, highlighting the need to repress translation of the mutated mRNA to promote genetic rescue (Markantone et al., 2018). An approach to specifically targeting *in organello* translation of human mitochondrial mRNAs has been developed recently (Cruz-Zaragoza et al., 2021). Chemically synthesized chimeras made of a precursor protein and a morpholino oligomer were shown to be efficiently imported into isolated human mitochondria, recognizing their target mRNA in the organelles and subsequently interfering with ribosome binding (Cruz-Zaragoza et al., 2021). However, such an approach relying on synthetic hybrid molecules seems difficult to be applied *in vivo*. Whether the protocol is able to discriminate between the mutated and non-mutated mRNAs on the basis of a single nucleotide difference and whether translation can be blocked anywhere in the mRNA remain to be established. We show here that the *trans*-cleaving hammerhead ribozymes (Persson et al., 2002; Val et al., 2011) function in human mitochondria, indicating that they find enough available Mg^2+^ to support their catalytic activity. Since *trans*-cleaving ribozymes can be designed against literally any sequence and adapted for most targets, our protocol can be applied to not only mutated organellar mRNAs but also tRNAs or rRNAs. The strategy can also be combined with allotopic expression of wild-type proteins or RNAs, native or with silent changes.

Using *trans*-cleaving ribozymes for target knockdown in mitochondria has further advantages over the organellar RNA interference and antisense strategies. Catalytic RNAs are autonomous, as they carry their own activity, while antisense RNAs or siRNAs only tag their target RNA and must rely on recruitment of available ribonucleases and degradation pathways. Also, for effective RNA interference, siRNAs need to include Ago2-binding sites for recognition (Gao et al., 2021), which imposes sequence constraints. Considering that most pathogenic mtDNA mutations are heteroplasmic, the well-designed *trans*-cleaving ribozymes should be able to discriminate between mutated and non-mutated RNAs bearing a single nucleotide difference and present at the same time in the organelles (Scherer and Rossi, 2003; Val et al., 2011). With target annealing arms providing a total of 15 base pairs (8 and 7), single mismatches should be sufficient to induce a drop in cleavage activity. Therefore, control of ribozyme specificity and discrimination of single nucleotide mismatches have been discussed on the basis of the cleavage mechanism (Birikh et al., 1997; Verma et al., 1997) and experimentally evaluated with different targets *in vitro* (Hertel et al., 1996; Kasai et al., 2002) and in human cells (Scherr et al., 1997). The TLS shuttles that we developed are novel for human mitochondria and have the advantage of being native sequences with innate ability for organellar uptake. They are likely to be translocated into mitochondria through the putative tRNA import route (Baleva et al., 2015). In addition, they do not contain the consensus internal promoter motifs of regular tRNA genes for RNA polymerase III (Geiduschek and Kassavetis, 2001) and thus can be easily included in customized constructs to be transcribed by RNA polymerase II. Moreover, the ribozyme cargo being translocated into mitochondria implies that BMV TLS and TMV TLS are not substrates for RNase P, the 5′-end processing enzyme of regular tRNA precursors (Lechner et al., 2015).

The expression of the human mtDNA and the balance of the mitochondrial transcriptome are under tight control. In addition to the exchange of small metabolic signals, multiple sets of ncRNAs participate in the intercompartment crosstalk (Dietrich et al., 2015; Weber-Lotfi and Dietrich, 2018; Huang et al., 2021). Remarkably, RNA transport through the mitochondrial membranes seems bidirectional (Giordani et al., 2021), and mtDNA-transcribed RNAs might be exported as well to other compartments, including imported nuclear-encoded lncRNAs like *SAMMSON* (Vendramin et al., 2018) and exported mtDNA-originating lncRNAs like *SncmtRNA* or *ASncmtRNA* (Burzio et al., 2009), bringing to a complex and dynamic organellar transcriptome. Our knockdown strategy using inducible transgene expression could particularly highlight rapid and early responses of the organellar transcriptome control and communication. Indeed, the anti-*ATP6* ribozyme level and the *ATP6* mRNA level are coordinated, i.e. the reconstitution of the *ATP6* pool starts immediately when the ribozyme level decreases (Figure 3; Supplementary Figures S5 and S6). Remarkably, the ATP6 protein level (Figure 6) shows the same kinetics as the *ATP6* mRNA level (Figure 3), implying rapid turnover of the protein and coupling between transcription and translation, different from the observation in early study where high stability of most mtDNA-encoded proteins masked the impact of mRNA knockdown triggered by mitochondrial RNA interference (Gao et al., 2021). Meanwhile, the transient knockdown is likely to reflect a high demand for mitochondrial activity in the transformants, possibly due to metabolic stimulation and cellular homeostasis re-adjustment upon tetracycline treatment for transgene induction. The drop in the *ATP6* mRNA level might be sensed, via the impairment of the OXPHOS chain activity, and trigger the transcription.

The data altogether establish that (i) BMV TLS and TMV TLS, but not TYMV TLS, can be translocated into the mitochondrial matrix in human cells, (ii) BMV TLS and TMV TLS can drive a 5′-end trailor RNA into the organelles in human cells, (iii) a *trans*-cleaving hammerhead ribozyme imported as a trailor RNA is functional in human mitochondria, and (iv) import of a functional *trans*-cleaving ribozyme enables specific cleavage and knockdown of a target mitochondrial mRNA and consequent knockdown of the corresponding protein, affecting mitochondrial OXPHOS function accordingly. By targeting mitochondrial gene expression at the transcriptional level, the approach allows further exploration of genetic regulatory pathways in human cells. The overall strategy brings novel views on tRNA and ncRNA import into human mitochondria, providing tools for analysing the control and communication of the human organellar genetic system, and contributes to developing gene therapy for mitochondrial diseases caused by mtDNA mutation.

## Materials and methods

Materials and methods are detailed in Supplementary material.

## Supplementary Material

mjad051_Supplemental_File

## Notes

**Author contributions:** P.G., R.V., and K.K. performed experiments and analysed the data. A.D. and K.R. developed and supervised the project. A.D. and K.R. interpreted the results. A.D. wrote the manuscript with the contribution of P.G. and K.R.
