# Supplementary material for: Mitochondrial transport of catalytic RNAs and targeting of the organellar transcriptome in human cells
Source: J Mol Cell Biol. 2023 Aug 17;15(8):mjad051. doi: 10.1093/jmcb/mjad051 (PMC11148835; doi:10.1093/jmcb/mjad051)
Supplement: mjad051_Supplemental_File [file mjad051_supplemental_file.pdf]

Mitochondrial transport of catalytic RNAs and targeting of the organellar transcriptome in human cells

Pawel Glodowicz, Konrad Kuczyński, Romain Val, André Dietrich, Katarzyna Rolle

Materials and Methods

Preparation of gene constructs

Three constructs were prepared for expression of the previously described PKTLS shuttle (Val et al., 2011) in human cells: i) the pcDNA5-135-PKTLS-cHDV construct encoding the PKTLS aminoacylatable with valine fused at the 3'-end to the sequence of the *Hepatitis delta virus* antigenomic *cis*-cleaving ribozyme (cHDV) (Perrotta and Been, 2007), ii) the pcDNA5-cHh-PKTLS-cHDV construct encoding the PKTLS aminoacylatable with valine fused at the 5'-end to a *cis*-cleaving hammerhead ribozyme (cHh) and at the 3'-end to the cHDV sequence and iii) the pcDNA5-cHh-PKTLSmet-cHDV construct encoding the PKTLS variant aminoacylatable with methionine (Dreher et al., 1996) fused at the 5'-end to a cHh and at the 3'-end to the cHDV sequence. The PKTLS-cHDV sequence was amplified by PCR from the pCK-PSTYPKTLScHDV plasmid (Val et al., 2011) with the AflII-PKTLS5P_F forward primer and the HDVr-BamHI_R reverse primer (Supplementary Table S1). The cHh-PKTLS-cHDV and cHh-PKTLSmet-cHDV sequences were amplified by PCR from the pCK-PSTYPKTLScHDV and pCK-SP72-PKTLSmet-HDV plasmids, respectively (Val et al., 2011) with the Hh5PKTLS_F forward primer and the HDVr-BamHI_R reverse primer (Supplementary Table S1). The AflII-PKTLS5P_F and Hh5PKTLS_F forward primers possessed a 5' *Afl*II restriction site, while the HDVr-BamHI_R reverse primer included a *Bam*HI restriction site. The Hh5PKTLS_F forward primer also contained the sequence of the cHh *cis*-hammerhead ribozyme. The obtained PCR products were cloned into the *Afl*II and *Bam*HI sites of the tetracycline-inducible transcription unit of the pcDNA5/FRT/TO vector (Invitrogen) and the resulting plasmids were used to transform human cell lines.

To prepare constructs suitable for *in vitro* expression, the BMV TLS and the TMV TLS sequences (*i.e*. the last 200 nucleotides at the 3'-end of the two viral genomes, accession numbers NC_002026/AB183261 and NC_001367/X68110, respectively) were amplified by PCR from corresponding viral cDNAs (a gift from David Gilmer and Salah Bouzoubaa, IBMP, Strasbourg) with the BrMVT7_F and ToMVT7_F direct primers containing the T7 RNA polymerase promoter and the BrMVBst_R and ToMVBst_R reverse primers containing a *Bst*NI restriction site (Supplementary Table S1). The obtained PCR products were directly cloned into the pUC19 vector (Promega). The resulting plasmids were linearized with the *Bst*NI enzyme and used for T7 RNA polymerase transcription.

The constructs expressing the anti-*ATP6 trans*-cleaving ribozymes associated with the BMV or TMV TLS were designed as above with an upstream *cis*-cleaving hammerhead ribozyme (cHh) and the downstream HDV *cis*-cleaving ribozyme (cHDV). The corresponding sequences were assembled by PCR amplification using as templates the above plasmids prepared for T7 RNA polymerase transcription, together with the large forward primers GUCRz_BMV_FW_3Rz, AUCRz_BMV_FW_3Rz, GUCRz_TMV_FW_3Rz, or AUCRz_TMV_FW_3Rz and the large reverse primers HDV_BMV_RV or HDV_TMV_RV (Supplementary Table S1). The forward primers included, from 5' to 3', a *Bam*HI site, the complete sequence of the relevant upstream *cis*-cleaving hammerhead ribozyme (cHh) followed by the anti-*ATP6 trans*-cleaving hammerhead ribozyme (GUCRz or AUCRz), a 40 nucleotide linker sequence and the first 24 nucleotides from the 5'-end of the BMV or TMV TLS. The reverse primers included, from 5' to 3', an *Xho*I site, the complement to the complete sequence of the HDV *cis*-cleaving ribozyme (cHDV) and the complement to the last 15 nucleotides from the 3'-end of the BMV or TMV TLS. The resulting PCR products

encoding the upstream *cis*-ribozyme, the anti-*ATP6 trans*-ribozyme, the linker, the TLS and the cHDV *cis*-ribozyme were directly cloned into the pGEM-T vector (Promega) for sequencing, re-excised with the *Bam*HI and *Xho*I restriction endonucleases, and cloned into the *Bam*HI and *Xho*I sites of the tetracycline-inducible transcription unit of the pcDNA5/FRT/TO vector (Invitrogen), yielding the pcDNA5-cHh-GUCRz-L-BMVTLS-cHDV, pcDNA5-cHh-AUCRz-L-BMVTLS-cHDV, pcDNA5-cHh-GUCRz-L-TMVTLS-cHDV and pcDNA5-cHh-AUCRz-L-TMVTLS-cHDV plasmids used to transform human cell lines.

Cell lines and transformation procedures

Flp-In T-REx 293 human kidney cells (R78007, Invitrogen; K650001 core kit, ThermoFischer) and HepG2 human cells (HB-8065, ATCC) were cultivated at 37ºC under air with 5% CO_2 in Dulbecco's Modified Eagle's Medium (DMEM, ATCC) and Eagle's Minimal Essential Medium (EMEM, Corning), respectively, supplemented with 10% (v/v) fetal bovine serum (FBS, EURx) and 1% (v/v) antibiotics solution [10 000 units penicillin, 10 mg streptomycin, 25 µg amphotericin B per ml (Sigma-Aldrich) for Flp-In T-REx 293 cells and 5 000 units penicillin, 5 mg streptomycin per ml (ThermoFischer) for HepG2 cells]. For assays in hypoxic conditions, stably transformed cell lines were cultured at 37°C under 5% CO_2 and 1% O_2.

Transfection of Flp-In T-REx 293 cells (80-90% confluence) with the pcDNA5-135-PKTLS-cHDV, pcDNA5-cHh-PKTLS-cHDV, pcDNA5-cHh-PKTLSmet-cHDV, pcDNA5-cHh-GUCRz-L-BMVTLS-cHDV, pcDNA5-cHh-AUCRz-L-BMVTLS-cHDV, pcDNA5-cHh-GUCRz-L-TMVTLS-cHDV and pcDNA5-cHh-AUCRz-L-TMVTLS-cHDV plasmids was carried out with Lipofectamine 2000 (Invitrogen). Genomic insertion of the construct in a transcriptionally active region was mediated by the Flp Recombinant Target (FLP) sequences present in both the pcDNA5/FRT/TO vector and a relevant locus in the nuclear genome. Recombination was ensured by the yeast Flp recombinase expressed from the pOG44 plasmid present in the Flp-In T-REx 293 cell line. Stably transformed cells were selected in culture medium supplemented with 50 µg/ml hygromycin B (ThermoFischer).

To prepare a Flp-In HepG2 cell line, parental HepG2 cells were plated into 6-well plates and cultured for 24 hours up to 85-90% confluence. On the next day, they were transfected with 1 µg of the pFRT/lacZeo plasmid (Invitrogen) using Lipofectamine 2000 (Invitrogen). Twenty four hours after transfection, the cells were replated on 60 mm dishes and subjected to selection with 150 µg/ml zeocin (ThermoFisher) for up to 3 weeks with regular medium change. To confirm transfection, a PCR reaction was performed with primers specific for the pFRT/lacZeo plasmid (Supplementary Table S1). Selected HepG2 cells carrying the pFRT/lacZeo plasmid were cultivated in 6-well plates up to 85-90% confluence and transfected with 1 µg of the pcDNA6/TR plasmid (Invitrogen), using Lipofectamine 2000. Twenty four hours after transfection, the cells were replated on 60 mm dishes and subjected to selection with 150 µg/ml zeocin (ThermoFisher) and 5 µg/ml blasticidin (Gibco) for up to 2 weeks. Transfection was confirmed by PCR using primers specific for the pcDNA6/TR plasmid (Supplementary Table S1). Finally, HepG2 cells carrying both the pFRT/lacZeo plasmid and the pcDNA6/TR plasmid were co-transfected with 1 µg of the pOG44 plasmid (Invitrogen) and 1 µg of the pcDNA5-cHh-GUCRz-L-BMVTLS-cHDV, pcDNA5-cHh-AUCRz-L-BMVTLS-cHDV, pcDNA5-cHh-GUCRz-L-TMVTLS-cHDV or pcDNA5-cHh-AUCRz-L-TMVTLS-cHDV plasmid. Flp-In HepG2 cells stably transformed with the different constructs were subsequently selected in culture medium supplemented with 100 µg/ml hygromycin B (ThermoFisher).

Induction of transgene expression

Transgenes in the pcDNA5/FRT/TO vector are under the control of the CMV/TetO2 tetracycline-inducible RNA polymerase II promoter. Tetracyline (from the K650001 core kit, ThermoFischer) was resuspended at 1 mg/ml in sterile water, filtered (0.22 µm filter) and stored at -20°C protected from light. Transgene expression was induced with tetracycline in

transformed cells at 80-90% confluence. Tetracycline concentrations ranging from 0.5 to 10 µg per ml culture medium were tested.

Preparation of labeled RNA substrates for *in vitro* organellar import

For mitochondrial import assays, radiolabed BMV TLS and TMV TLS transcripts were generated by *in vitro* transcription of the above *Bst*NI-linearized constructs in the presence of [α-^{32}P]UTP. Transcription reactions were performed using a MEGAshortscript T7 Kit (ThermoFischer) as described by the manufacturer. Reaction products were purified using the NucAway Spin Column kit (ThermoFischer) according to the recommended protocol.

Preparation of Human Import Directing Proteins (HmIDPs)

HmIDP fractions were prepared essentially according to Entelis et al. (2001). HepG2 cells were harvested with a trypsin solution [0.025% (w/v) trypsin, 0.01% (w/v) EDTA in phosphate-buffered saline (PBS), ThermoFischer], washed with PBS, suspended in NPMD buffer (20 mM Na-phosphate buffer, pH 6.5, 150 mM NaCl, 1 mM $MgCl_2$, 5 mM DTT) containing a cocktail of protease inhibitors (cOmplete Mini, EDTA-free, Roche) and disrupted by sonication (4 times 60 sec at maximal frequency). Cellular debris were removed by centrifugation at 4000 *g* for 10 min. To eliminate nucleic acids, polyethyleneimine [0.8 µL of a 50% (w/v) aqueous solution of polyethyleneimine per 100 µg of initial cellular material] was added. The precipitate formed was removed by centrifugation at 20 000 *g* for 10 min. Proteins were fractionated by differential ammonium sulfate precipitation and fractions precipitating at 30, 60, 90% of saturation were dialyzed against NPMD buffer containing 50% (v/v) of glycerol.

Isolation of mitochondria

Tetracycline-induced transgenic cells or standard HepG2 cells grown to 90-95% confluence were harvested with a trypsin solution [0.025% (w/v) trypsin, 0.01% (w/v) EDTA in phosphate-buffered saline (PBS), ThermoFischer] and washed with PBS. Mitochondria were subsequently isolated using a Mitochondria Isolation Kit (Mitochondria MidiMACS Starting Kit, Miltenyl Biotec) according to the manufacturer's protocol. Mitoplasts were generated by hypotonic shock. The mitochondrial pellet was resuspended in 100 µL of breakage buffer [BB; 0.6 M mannitol, 1 mM EDTA, 10 mM Na-PIPES, pH 6.7, 0.3% (w/v) BSA] and diluted 10 times in 10 mM HEPES, pH 6.8. After 10 min incubation on ice, sucrose was added up to 0.25 M. The resulting mitoplasts were harvested by centrifugation for 5 min at 10 000 *g* and washed twice with BB. Residual cytosolic RNA contamination was removed by addition of a mixture of nucleases (10 units/ml of micrococcal nuclease, 100 µg/ml of RNase A and 25 units/ml of phosphodiesterase) and incubation for 15 min at 37°C. Nuclease treatment was followed by an incubation of the organelles at 20°C for 5 min in the presence of 1 mM $CaCl_2$ and the mitochondrial RNA was finally isolated.

Alternatively, cells were resuspended in homogenization buffer [mannitol 0,6 M, Tris-HCl (pH 7,4) 10 mM, EGTA 10 mM, BSA 0.1% (w/v)] and lyzed in a Dounce homogenizer. The suspension was centrifuged successively at 600 *g* for 5 min at 4°C and 11 000 *g* for 10 min at 4°C. The final pellet was resuspended in 500 µL of homogenization buffer containing 50 µg RNase A and 375 units RNase T1. After 15 min incubation at room temperature, 500 µL of homogenization buffer, 1 µL of phenylmethylsulfonyl fluoride (PMSF, 0.1 M) and 1 µL of proteinase K (10 mg/ml) were added prior to 30 min incubation on ice. Mitochondria were subsequently re-isolated on a discontinuous Percoll gradient [13,5%, 21%, 45% (v/v)] and washed with homogenization buffer without BSA completed with 10 mM EGTA and 10 mM EDTA. To prepare mitoplasts, mitochondria were suspended in 500 µL of homogenization buffer and digitonine (170 µg/mg mitochondrial proteins) was added. After 15 min incubation on ice, mitoplasts were recovered by centrifugation at 11 000 *g* for 15 min at 4°C and washed with homogenization buffer without BSA before treatment with RNases A and T1.

In vitro organellar import

Import assays were carried out essentially as described previously (Entelis et al., 2001). The reaction mixture (100 µL) containing mitochondria isolated from standard HepG2 cells (50 µg protein), radiolabeled RNA substrate (3 pmol) and HmIDP protein extract (10 µg protein) in import buffer [0.44 M mannitol, 20 mM HEPES-KOH (pH 6.8), 20 mM KCl, 2.5 mM $MgCl_2$, 1 mM ATP, 5 mM DTT, 0.5 mM PMSF, 0.1 mM diisopropyl fluorophosphate (DIFP), 0.1 mM L-lysine, 0.5 mM phosphoenol pyruvate, 4 units of pyruvate kinase] was incubated at 30°C for 20 min. Subsequently, unimported RNA was removed by adding nucleases (10 units/ml micrococcal nuclease, 100 µg/ml RNase A, 25 units/ml phosphodiesterase) to the mixture and further incubating the assay at 20°C for 5 min in the presence of 1 mM $CaCl_2$. The suspension was then diluted 5-fold with buffer BB [mannitol 0.6 M, EDTA 1 mM, NaPIPES (pH 6.7) 10 mM, BSA 0.3% (w/v)] and centrifuged at 14 000 *g* for 10 min at 4°C. The obtained mitochondrial pellet was resuspended in 100 µL of buffer BB. One half of the mitochondria was pelleted again and extracted for direct analysis, while the other half was diluted 10-fold in 10 mM HEPES-KOH, pH 6.8, to disrupt the outer membrane. After 10 min incubation on ice, 0.25 M sucrose was added and the suspension was centrifuged at 14 000 *g* for 10 min at 4°C. The pellet was washed twice with BB buffer. Mitochondria and mitoplasts were lyzed for 1 min at 100°C in 1% (w/v) SDS, 0.1 M sodium acetate, 0.05% (w/v) diethyl pyrocarbonate. The RNA was finally isolated by guanidinium thiocyanate-phenol-chloroform extraction (Chomczynski and Sacchi, 2006), separated on a 10% (w/v) polyacrylamide gel and analyzed by autoradiography.

RNA extraction and RT-qPCR analyses

RNA was extracted from whole cells and mitochondrial preparations according to standard TRI Reagent protocols (Invitrogen). Remaining DNA was digested with DNase I using the DNA Free reagent kit (ThermoFischer) as described by the manufacturer.

Reverse transcription was carried out with the Transcriptor First Strand cDNA Synthesis Kit (Roche) according to the manufacturer's protocol. RNA sample quality and absence of remaining DNA was assessed through standard RT-PCR reactions and the amplification products were analyzed on agarose gel. RT-real-time PCR was subsequently run on a CFX Connect thermal cycler (Bio-Rad). Reaction mixtures were prepared with the LightCycler 480 SYBR Green I Master reagent Kit (Roche) according to manufacturer's protocols. As reference genes we used those for actin beta (ACTB), glyceraldehyde 3-phosphate dehydrogenase (GAPDH), hypoxanthine-guanine phosphoribosyl transferase 1 (HPRT1) and porphobilinogen deaminase (PBDG). Analyses in relation to the reference genes was performed by the relative quantification method (ΔΔCt method). Three independent biological replicates were analyzed.

FLOE

For fluorescently labeled oligonucleotide extension assays (FLOE, Lloyd et al., 2005), reverse transcription was run with 5 µg of mitochondrial RNA and 5 nmol of the atp6_atp8_FAM reverse primer (Supplementary Table S1) labeled at the 5'-end with 6-Carboxyfluorescein (6-FAM) using the Transcriptor First Strand cDNA Synthesis Kit (Roche). After RNase A digestion, the cDNAs were ethanol precipitated, redissolved in water and completed with 0.5 µL of GeneScan 1200 LIZ dye Size Standard (Applied Biosystems). Samples (10 µL final volume) were finally analyzed with a capillary electrophoresis sequencer (Applied Biosystems).

Western blot analyses

Transgenic lines were grown in 6-well plates to a confluence of 85-90% and treated at Day 0 with 10 µg/ml tetracycline for induction of transgene expression. Cell samples were

subsequently taken every day until Day 6 and lysed by sonication in 10 mM Tris-HCl, pH 7, containing a cocktail of protease inhibitors (cOmplete Mini, EDTA-free, Roche). The lysate was centrifuged at 15 000 *g* for 10 min and the supernatant was transferred to new tubes. Protein samples (40 µg) were denatured, separated by SDS-PAGE and transferred to polyvinylidene fluoride (PVDF) membranes following standard protocols. Membranes were blocked with 5% (w/v) skimmed milk. After incubation with primary and secondary antibodies, proteins of interest were detected with the SuperSignal West Pico PLUS Chemiluminescent Substrate (Life Technologies). The MT-ATP6 protein was detected with polyclonal antibodies (Life Technologies) at a 1:500 dilution. The MT-COX1 and MT-COX2 proteins were detected with monoclonal antibodies (Cell Signaling Technology) at a 1:1000 (COX1) or 1:750 (COX2) dilution. As a control, the GAPDH protein was detected with the GAPDH 0411 monoclonal antibody (Santa Cruz Biotechnology) at a 1:500 dilution. The peroxydase-conjugated anti-rabbit A6154 antibody (Sigma-Aldrich) was used as a secondary antibody at a 1:10000 dilution. Antibodies were diluted in 5% (w/v) skimmed milk. The intensity of individual bands was analyzed quantitatively with the Multi Gauge ver. 2.0 software (Fujifilm). The relative level of MT-ATP6, MT-COX1 and MT-COX2 protein was normalized against the GAPDH level.

Cytotoxicity assays

For cytotoxicity assays, cells were grown in 96-well plates to a confluence of 85-90% and treated with 0.5 to 10 µg/ml tetracycline. After 24 h, the culture medium was withdrawn and 100 µL of MTT [3-(4,5-dimethylthiazol-2-yl)-2,5-diphenyltetrazolium bromide] at 5 mg/ml in EMEM medium were added to each well. The plates were incubated for 90 min at 37°C. The MTT solution was then withdrawn and 100 µL of DMSO (dimethyl sulfoxide) were added per well, followed by shaking for 10 min at 230 rpm to evenly dissolve the formazan generated. The amount of MTT reduced to the purple formazan was finally measured spectrophotometrically at a wavelength of 590 nm using a BioTek Synergy Microplate Reader.

Mitochondrial membrane potential (ΔΨm) analysis

The JC-1 cationic carbocyanine dye was used as a probe to evaluate the mitochondrial membrane potential status in whole cells. In a regular physiological state, the JC-1 dye is driven into mitochondria along the membrane potential and aggregates in the organelles. The so-called J-aggregate form can be quantified by reading the corresponding emission of red fluorescence at 590 nm. In cells with an impaired mitochondrial membrane potential, the JC-1 dye remains in its monomeric form in the cytosol and emits green fluorescence at 529 nm. Fluorescence of both the aggregated and the monomeric form can be triggered by light with a wavelength of 514 nm. Carbonyl cyanide m-chlorophenyl hydrazine (CCCP) was used as a positive control. CCCP acts as a ionophore and directly dissipates the potential of the mitochondrial membrane. Cells treated with CCCP should thus fluoresce only in the range of 529 nm.

Stably transformed cells were grown in 96-well plates to a confluence of 85-90% and treated at Day 0 with 10 µg/ml tetracycline for transgene induction. Test samples were subsequently generated every day. Day 0, before transgene expression, was considered as the regular physiological state. For each time point, the culture medium was withdrawn and replaced by 100 µL of DMEM (for Flp-In T-REx cells) or EMEM (for Flp-In HepG2 cells) medium complemented with 2 µM of JC-1 dye (ThermoFischer). The plates were subsequently incubated for 30 min at 37°C and the medium was replaced by 100 µL of warm PBS per well. Total fluorescence at 529±5 nm and 590±5 nm was measured using a fluorescence reader. Then, a representative sample of the measured wells was imaged using a confocal microscope in the same wavelength range. Double reading was used to eliminate false readings caused by poor condition of the cells.

Analysis of energy metabolism

Activity of the OXPHOS chain was evaluated with an Agilent Seahorse XFp analyzer using a Seahorse XF Cell Mito Stress Test Kit following the instructions of the manufacturer. Built-in injection ports on the XFp sensor cartridges allow to add OXPHOS modulators into the wells carrying the cells to reveal the key parameters of mitochondrial function. The modulators included in the kit are Oligomycin, Carbonyl cyanide-4 (trifluoromethoxy) phenylhydrazone (FCCP), Rotenone and Antimycin A. They are injected into the wells in this order.

To run the assays, stably transformed cell lines were seeded in Agilent XFp 8-well cell culture microplates (7.5×10^3 cells in 100 µl medium per well). At 80-90% confluence, transgene expression was induced with 10 µg/ml tetracycline and the oxygen consumption rate (OCR) of the cells was tested in the absence and presence of the above mentioned effectors. Briefly, the culture medium was removed from each well and replaced with 180 µL of Seahorse XF Base Medium containing 1 mM pyruvate and 2 mM glutamine, supplemented with 10 mM glucose and prewarmed to 37°C. The cells were incubated in a CO_2-free incubator at 37°C for 1 h. Prior to measurements, the XFp analyzer gently mixed the assay media in each well for 10 min to allow the oxygen partial pressure to reach equilibrium. The OCR and the extracellular acidification rate (ECAR) were measured simultaneously three times to establish a baseline rate. The OCR of the samples was tested over 7 min time intervals. The Agilent Seahorse XF Cell Mito Stress Test Report Generator automatically calculates the Agilent Seahorse XFp Cell Mito Stress Test parameters from Wave data that have been exported into MS Excel.

Statistical analyses

Results represent mean values of three independent biological replicates ± standard deviation (SD). Statistical significance was evaluated using the GraphPad Prism ver. 5.1 (GraphPad) software. Differences between the mean values of the test and the control samples were evaluated using ANOVA variance extended by Tukey or Bonferroni post hoc tests. Statistically significant results were assigned as: * for $p<0.05$; ** for $p<0.01$; *** for $p<0.001$; no statistical significance for $p\geq0.05$.

Supplementary Results

The TYMV PKTLS tRNA-like shuttle is unable to translocate into the mitochondrial matrix in human cells

In line with our previous success in plants (Val et al., 2011; Sultan et al., 2016; Niazi et al., 2019), we tested the possiblity to translocate the TYMV PKTLS (*i.e.* the last 120 nucleotides at the 3'-end of the viral genomic RNA) into mitochondria in human cells. The corresponding sequence was assembled with the HDV *cis*-ribozyme sequence (Perrotta and Been, 2007) (cHDV) at its 3'-end in the pcDNA5-135-PKTLS-cHDV plasmid under the RNA polymerase II promoter of the pcDNA5/FRT/TO vector (see Materials and Methods). Flp-In T-REx 293 human kidney cells stably transformed with this plasmid were generated. Upon expression of a PKTLS-cHDV transcript mediated by RNA polymerase II, self-cleaving of the HDV *cis*-ribozyme was expected to eliminate all downstream vector sequences together with the polyA tail. Conversely, due to the location of the polymerase II transcription start site with respect to the cloning site of the transgene in the construct, 135 nucleotides of vector sequence were added as a cargo to the 5'-end of the PKTLS in the transcripts generated (Supplementary Figure S1A). This cargo sequence however was not coding and carried no function in the cells.

The possibility to express the TYMV PKTLS, *i.e.* a plant viral sequence, in human cells was assessed by RT-PCR upon transgene induction with tetracycline. Mitochondria were subsequently isolated from cells at 24 h after induction. Half of the organelles was directly

used for further processing, while the other half was submitted to digitonine treatment, so as to generate mitoplasts by breaking the outer membrane and exposing RNAs that would be stuck in the intermembrane space. Both organelle fractions were submitted to RNase treatment prior to lysis and RNA extraction. RT-PCR analysis with specific primers (Supplementary Table S1) highlighted the presence of the PKTLS RNA in intact mitochondria, but not in mitoplasts (Supplementary Figure S1B). Conversely, the mitochondrial *ND3* control mRNA was present in both fractions, assessing that the matrix material was not lost during mitoplast preparation. The TYMV PKTLS thus appeared to be able to translocate through the mitochondrial outer membrane, but unable to cross the inner membrane in human cells.

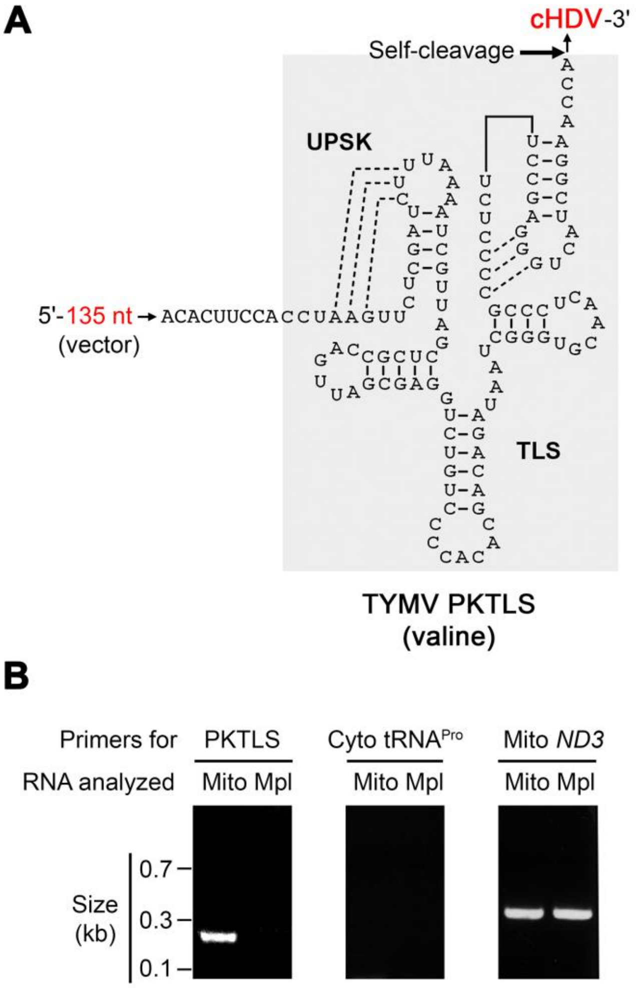

Supplementary Figure S1 Mitochondrial translocation of the TYMV PKTLS in Flp-In T-REx 293 human kidney cells stably transformed with the pcDNA5-135-PKTLS-cHDV construct. **A** Schematic representation of the primary transcript deriving from the transgene. The final product carries 135 nucleotides of upstream vector sequence, while self-cleaving of the cHDV ribozyme eliminates downstream vector sequences and releases the PKTLS 3'-CCA end. **B** RT-PCR analysis of RNAs extracted from mitochondria (Mito) or mitoplasts (Mpl). RNAs were probed for the TYMV PKTLS (PKTLS), the cytosol-specific $tRNA^{Pro}$ (Cyto $tRNA^{Pro}$) and the mitochondrial *ND3* mRNA (Mito *ND3*). RT-PCR products were fractionated on agarose gel.

Following these observations, we tested two adaptations of our strategy. The first idea was to test whether the presence of the 135 nucleotide cargo sequence at the 5'-end of the PKTLS moiety was preventing final translocation through the inner membrane. In this respect, we included in a second construct called pcDNA5-cHh-PKTLS-cHDV, a *cis*-cleaving hammerhead ribozyme (cHh) upstream of the PKTLS sequence, in addition to the downstream cHDV, so that after transcription and self-cleavage of the upstream and

downstream ribozymes the final product was expected to be the PKTLS moiety alone (Supplementary Figure S2A). The *cis*-cleaving hammerhead ribozyme was designed according to Fechter et al. (1998).

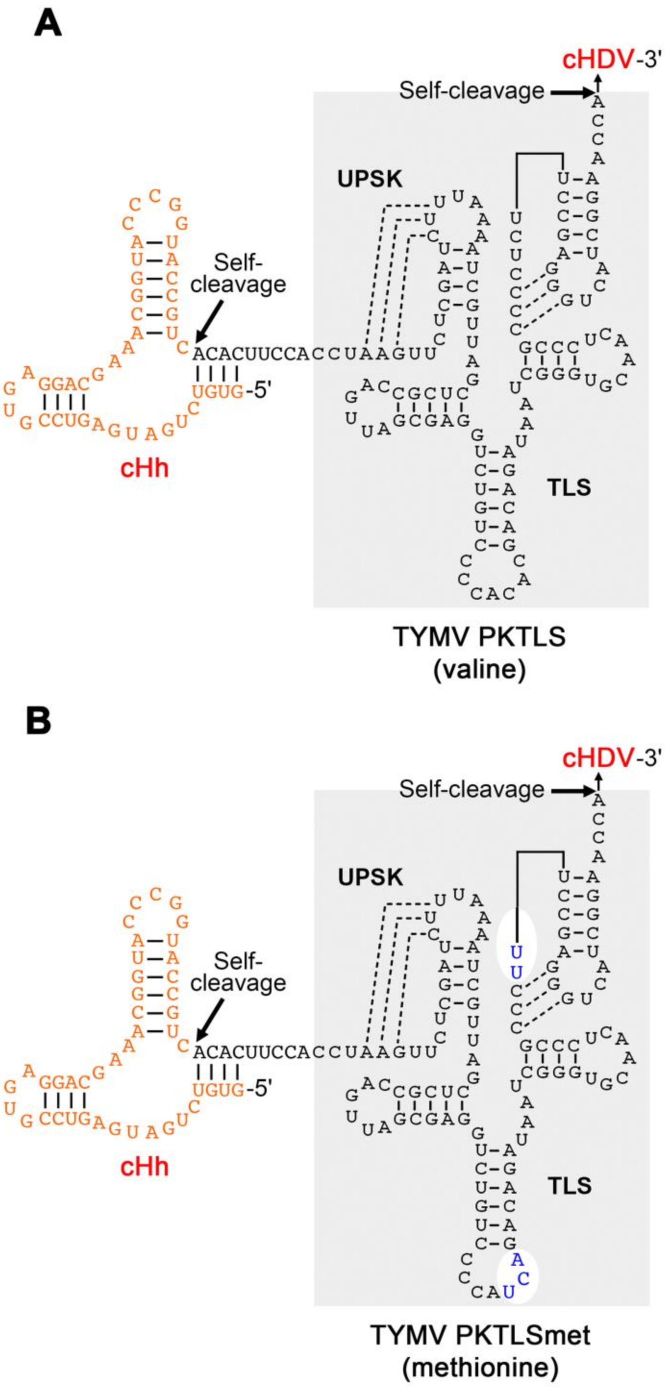

Supplementary Figure S2 Schematic representation of the primary transcript deriving from the transgene in Flp-In T-REx 293 human kidney cells stably transformed with the pcDNA5-cHh-PKTLS-cHDV construct (**A**) or the pcDNA5-cHh-PKTLSmet-cHDV construct (**B**). The final products are restricted to the PKTLS (**A**) or the PKTLSmet (**B**) sequence upon self-cleaving of the 5' cHh ribozyme and the 3' cHDV ribozyme. Nucleotide mutations switching the aminoacylation specificity from valine to methionine are indicated in blue on a white background (**B**).

As mentioned above, little information is available on the set of tRNAs capable of translocation into human mitochondria. The second option was thus to change the aminoacylation specificity of the TYMV PKTLS moiety. A TYMV PKTLS variant with methionine specificity has been described earlier, upon mutations in the acceptor stem

pseudoknot and anticodon switch from CAC to CAU (Dreher et al., 1996). This PKTLSmet variant served as negative organellar import control in our previous studies in plants (Val et al., 2011), as cytosolic $tRNA^{Met}$ is not found in plant mitochondria (Salinas et al. 2008). Whether methionine specificity can provide importability into human mitochondria has not been documented. We tested such a possibility with the pcDNA5-cHh-PKTLSmet-cHDV construct encoding the TYMV PKTLSmet variant flanked with an upstream hammerhead *cis*-ribozyme and with the downstream HDV *cis*-ribozyme (Supplementary Figure S2B). The pcDNA5-cHh-PKTLS-cHDV construct and the pcDNA5-cHh-PKTLSmet-cHDV construct were both properly expressed in stably transformed Flp-In T-REx 293 cells and their products were recovered in intact mitochondria, but neither the valine-specific PKTLS without a 5' cargo nor the PKTLSmet variant was detected in mitoplast fractions.

BMV and TMV tRNA-like structures are taken up by isolated human mitochondria *in vitro*

Failure to obtain complete translocation of the valine-specific TYMV PKTLS, or of its methionine accepting mutant derivative, into mitochondria in human cells prompted us to test other plant viral TLSs with further aminoacylation specificities. Apart from valine, two other native aminoacylation specificities have been documented for plant viral TLSs, *i.e.* histidine and tyrosine (Mans et al., 1991). The BMV TLS is a representative of the tyrosine-accepting group, while the TMV TLS is a histidine-accepting representative and we decided to evaluate the possibility to use one or the other of them as an organellar shuttle in human cells.

As a first approach, we tested whether the BMV or TMV TLS can be taken up by isolated human mitochondria. To that end, the last 200 nucleotides from the 3'-end of each genomic RNA were amplified by PCR from BMV and TMV cDNAs (a gift from David Gilmer and Salah Bouzoubaa, IBMP, Strasbourg) and cloned under the control of the T7 RNA polymerase promoter. A *Bst*NI restriction site was placed at the 3' end of the TLS sequences. The resulting templates linearized with *Bst*NI were transcribed with T7 RNA polymerase in the presence of [α-^{32}P]UTP, yielding radiolabeled BMV and TMV TLS with a regular CCA end. Both were used for *in vitro* import assays with mitochondria isolated from standard HepG2 human cells. Importing tRNAs into human mitochondria *in vitro* needs, among other factors, the presence of import-directing proteins (Entelis et al., 2001). Human import directing proteins (HmIDPs) were prepared, also from HepG2 cells, and separated into fractions precipitating at 30, 60 and 90% ammonium sulfate saturation. Following the import step, one part of the mitochondria was analyzed directly, while mitoplasts were generated from the other part. In these assays, the BMV TLS and the TMV TLS were both recovered in the final RNA fractions from mitochondria and from mitoplasts (Supplementary Figure S3), implying that they can both be taken up by isolated human organelles. All three HmIDP fractions stimulated the import (Supplementary Figure S3).

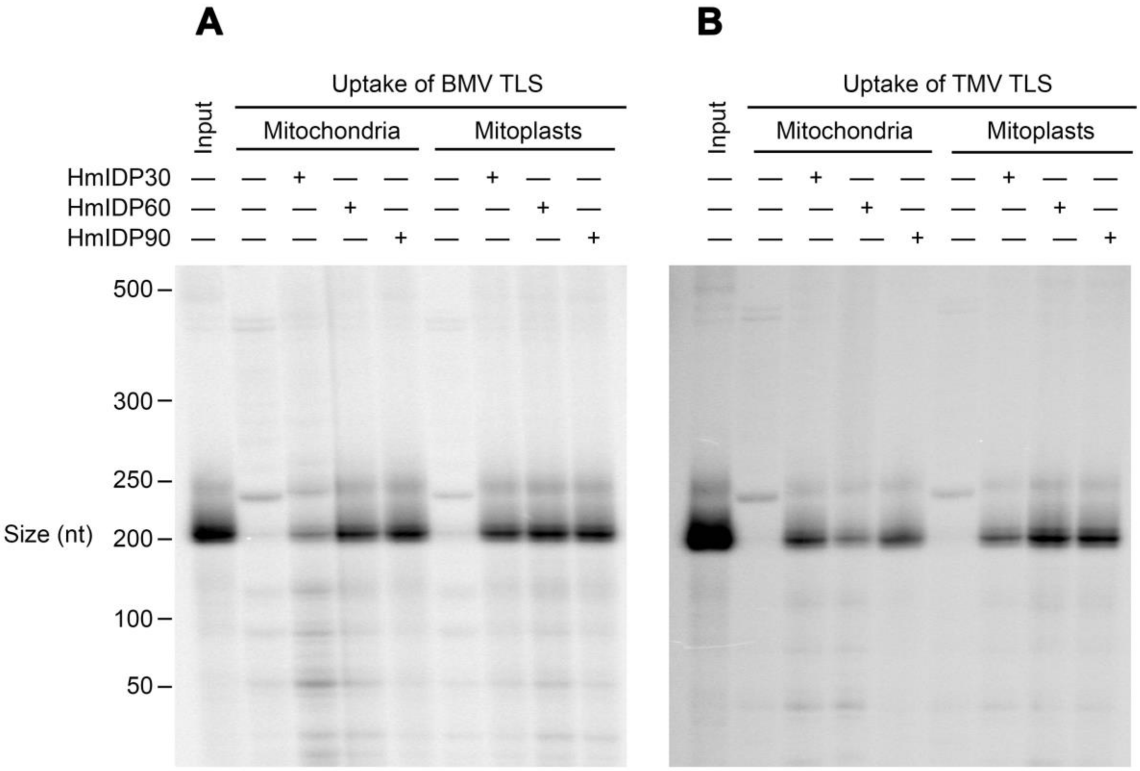

Supplementary Figure S3 *In vitro* import of BMV and TMV tRNA-like structures into isolated human mitochondria. *In vitro* synthesized [^{32}P]-radiolabeled BMV TLS (**A**) and TMV TLS (**B**) were incubated with isolated mitochondria from HepG2 cells in the presence of fractions of human import directing proteins (HmIDPs) precipitating at 30% (HmIDP30), 60% (HmIDP60) or 90% (HmIDP90) ammonium sulfate saturation. After the import step, one half of the mitochondria was kept intact, while the other half was treated so as to generate mitoplasts. Nucleic acids were extracted from both samples. RNAs were finally fractionated by electrophoresis on polyacrylamide gels and revealed by autoradiography. Samples of the [^{32}P]-radiolabeled transcripts used as import substrates were also loaded on the gels (Input). The pictures are representative of three independent experiments.

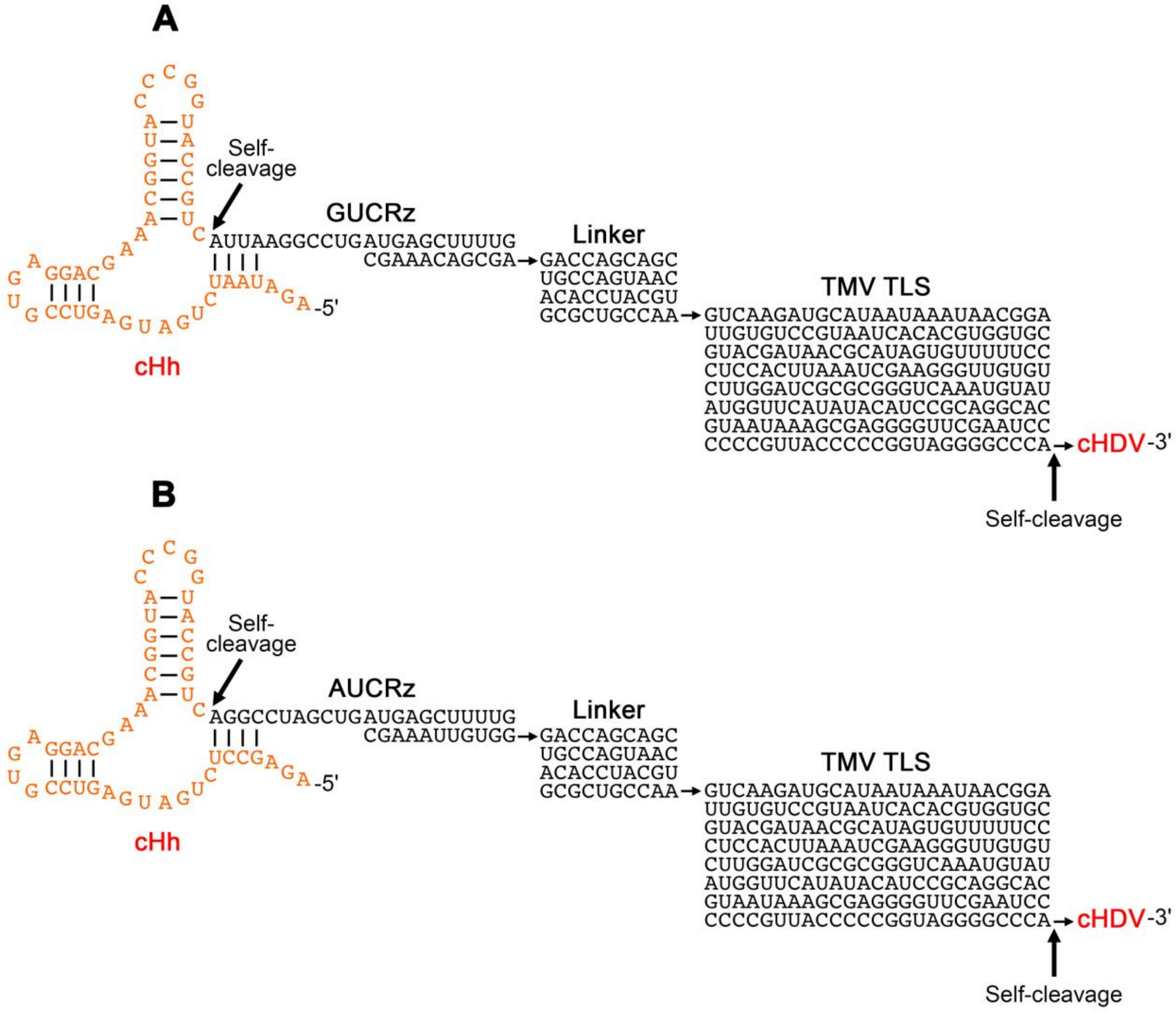

Supplementary Figure S4 Schematic representation of the primary transcript deriving from the transgene in human cells stably transformed with the pcDNA5-cHh-GUCRz-L-TMVTLS-cHDV construct (**A**) or the pcDNA5-cHh-AUCRz-L-TMVTLS-cHDV construct (**B**). Following self-cleaving of the 5' cHh ribozyme and the 3' cHDV ribozyme, the final products contain the GUCRz *trans*-ribozyme (**A**) or the AUCRz *trans*-ribozyme (**B**) sequence combined with the TMV TLS through the linker L.

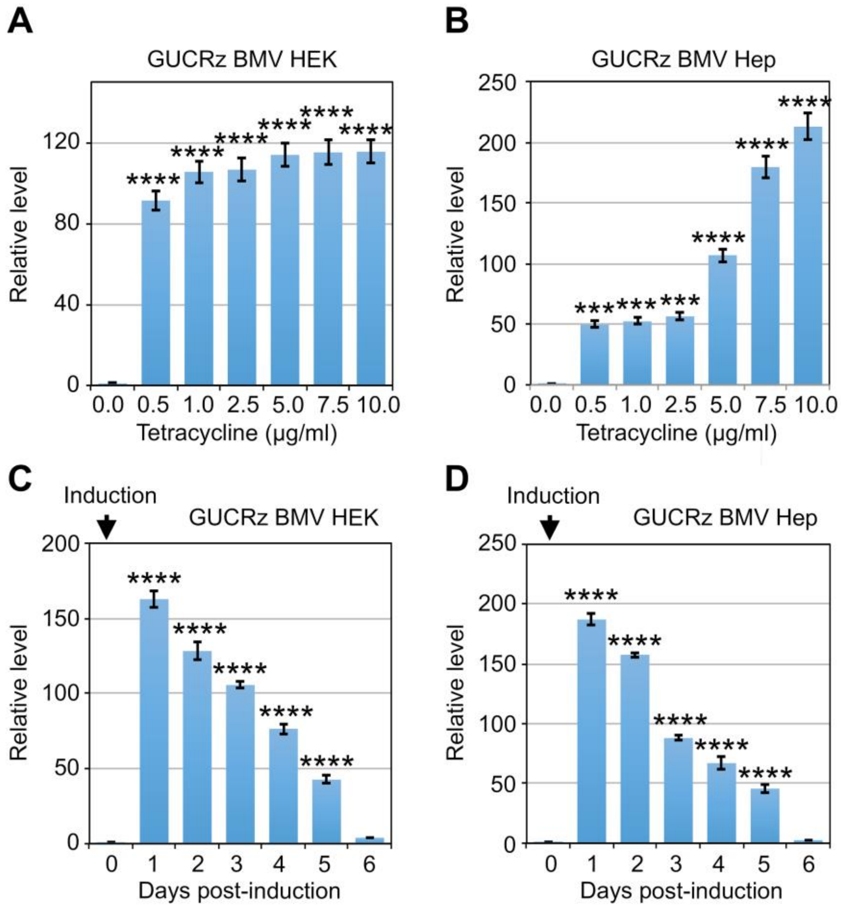

Supplementary Figure S5 Expression of the GUCRz-L-BMV TLS chimeric RNA (GUCRz-BMV) in HEK (**A** and **C**) and Hep (**B** and **D**) cells stably transformed with the pcDNA5-cHh-GUCRz-L-BMVTLS-cHDV construct. **A** and **B** Transgene expression was induced at Day 0 with increasing concentrations of tetracycline and cells were sampled at Day 1. **C** and **D** Transgene expression was induced with 10 μg/ml tetracycline at Day 0 and samples were subsequently taken every day until Day 6. Total RNA was extracted from all samples and analyzed by RT-qPCR. Data from three independent biological replicates were analyzed with the Student's *t*-test; * = $p < 0.05$; ** = $p < 0.01$; *** = $p < 0.001$; **** = $p < 0.0001$. Error bars represent ± SD.

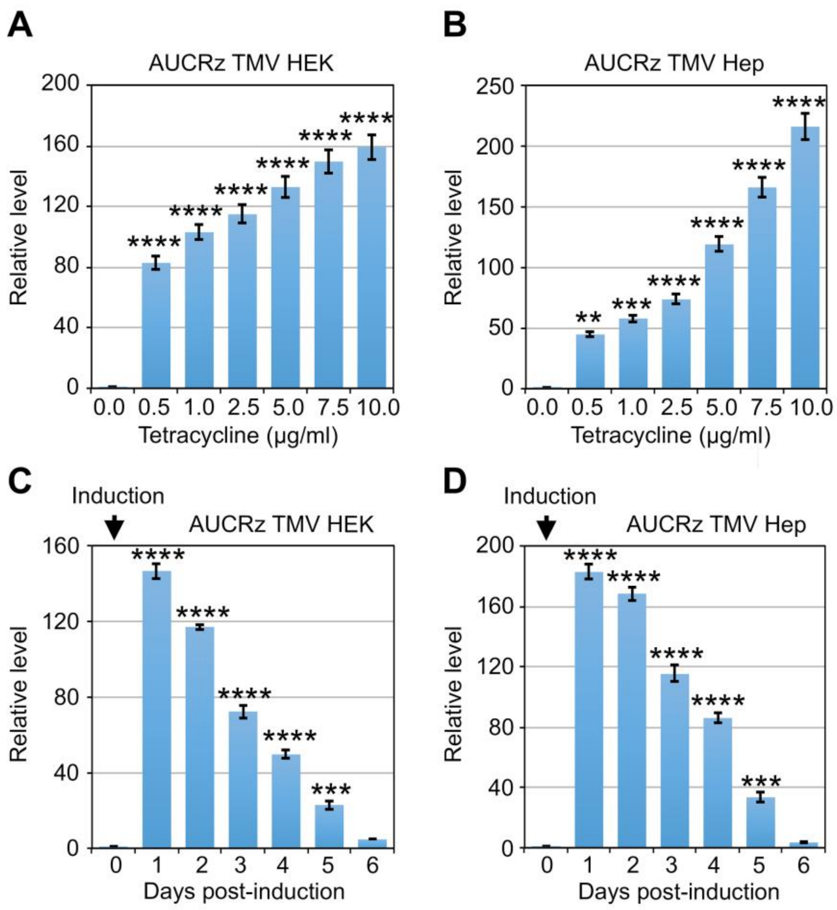

Supplementary Figure S6. Expression of the AUCRz-L-TMV TLS chimeric RNA (AUCRz-TMV) in HEK (**A** and **C**) and Hep (**B** and **D**) cells stably transformed with the pcDNA5-cHh-AUCRz-L-TMVTLS-cHDV plasmid. **A** and **B** Transgene expression was induced at Day 0 with increasing concentrations of tetracycline and cells were sampled at Day 1. **C** and **D** Transgene expression was induced with 10 µg/ml tetracycline at Day 0 and samples were subsequently taken every day until Day 6. Total RNA was extracted from all samples and analyzed by RT-qPCR. Data from three independent biological replicates were analyzed with the Student's *t*-test; * = $p < 0.05$; ** = $p < 0.01$; *** = $p < 0.001$; **** = $p < 0.0001$. Error bars represent ± SD.

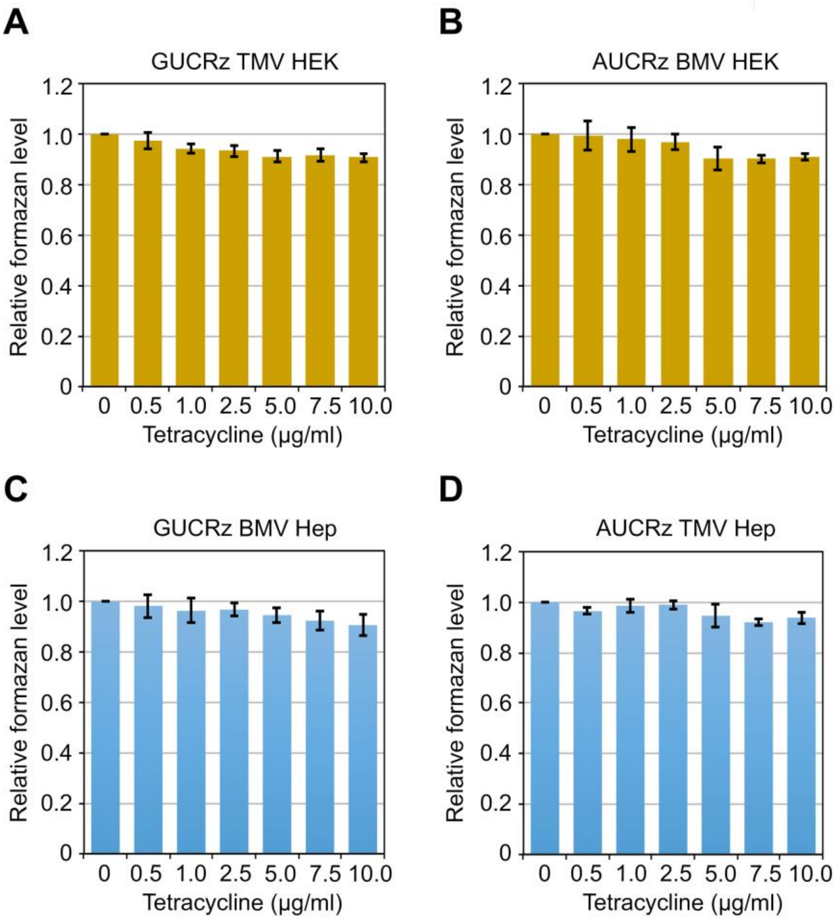

Supplementary Figure S7. Cytotoxicity tests. Transgene expression was induced at Day 0 with increasing concentrations of tetracycline in HEK (**A** and **B**) and Hep (**C** and **D**) human cells stably transformed with the pcDNA5-cHh-GUCRz-L-TMVTLS-cHDV (**A**), the pcDNA5-cHh-AUCRz-L-BMVTLS-cHDV (**B**), the pcDNA5-cHh-GUCRz-L-BMVTLS-cHDV (**C**) or the pcDNA5-cHh-AUCRz-L-TMVTLS-cHDV (**D**) plasmid. Cells were sampled at Day 1 and submitted to an MTT cytotoxicity test (see Materials and Methods). The level of formazan generated by alive cells was measured spectrophotometrically at a wavelength of 590 nm. All graphs are based on the results of six independent assays. Student's *t*-test analyses revealed no statistically significant differences. Error bars represent ± SD.

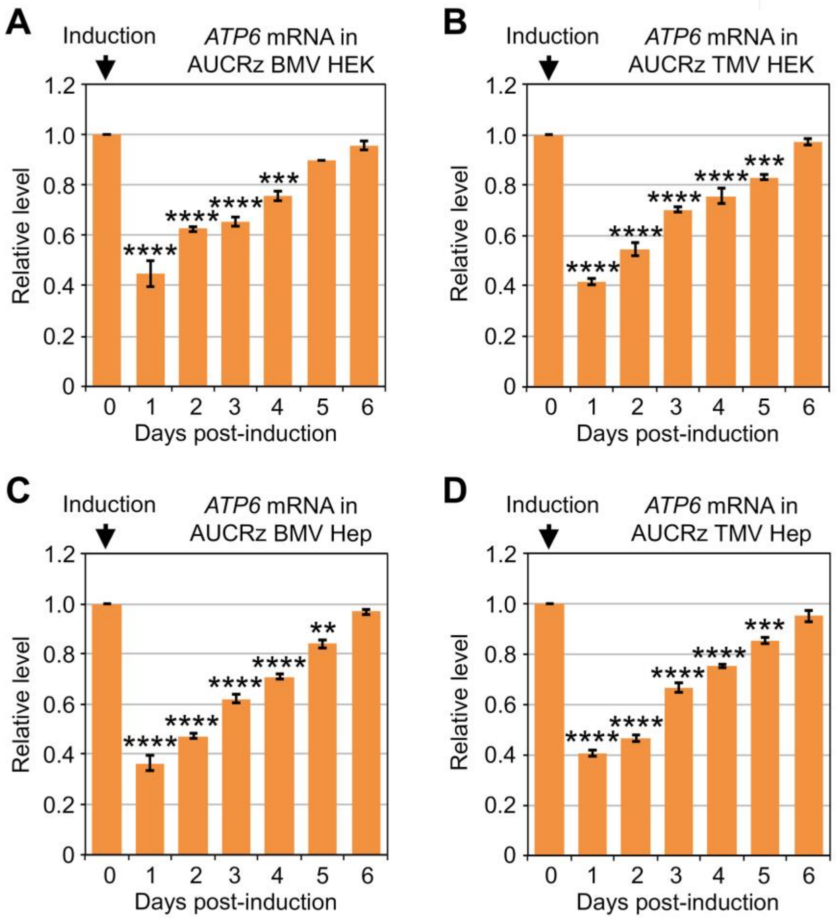

Supplementary Figure S8. Expression of the AUCRz-L-BMV TLS or AUCRz-L-TMV TLS chimeric RNA in HEK (**A** and **B**) or Hep (**C** and **D**) transformants results in mitochondrial target RNA knockdown. Transgene expression was induced at Day 0 with 10 µg/ml of tetracycline in cells stably transformed with the pcDNA5-cHh-AUCRz-L-BMVTLS-cHDV (**A** and **C**, AUCRz BMV) or pcDNA5-cHh-AUCRz-L-TMVTLS-cHDV (**B** and **D**, AUCRz TMV) plasmid and samples were subsequently taken every day until Day 6. Total RNA was extracted from all samples and steady-state levels of the mitochondrial *ATP6* mRNA were analyzed by RT-qPCR. Data from three independent biological replicates were analyzed with the Student's *t*-test; * = $p < 0.05$; ** = $p < 0.01$; *** = $p < 0.001$; **** = $p < 0.0001$. Error bars represent ± SD.

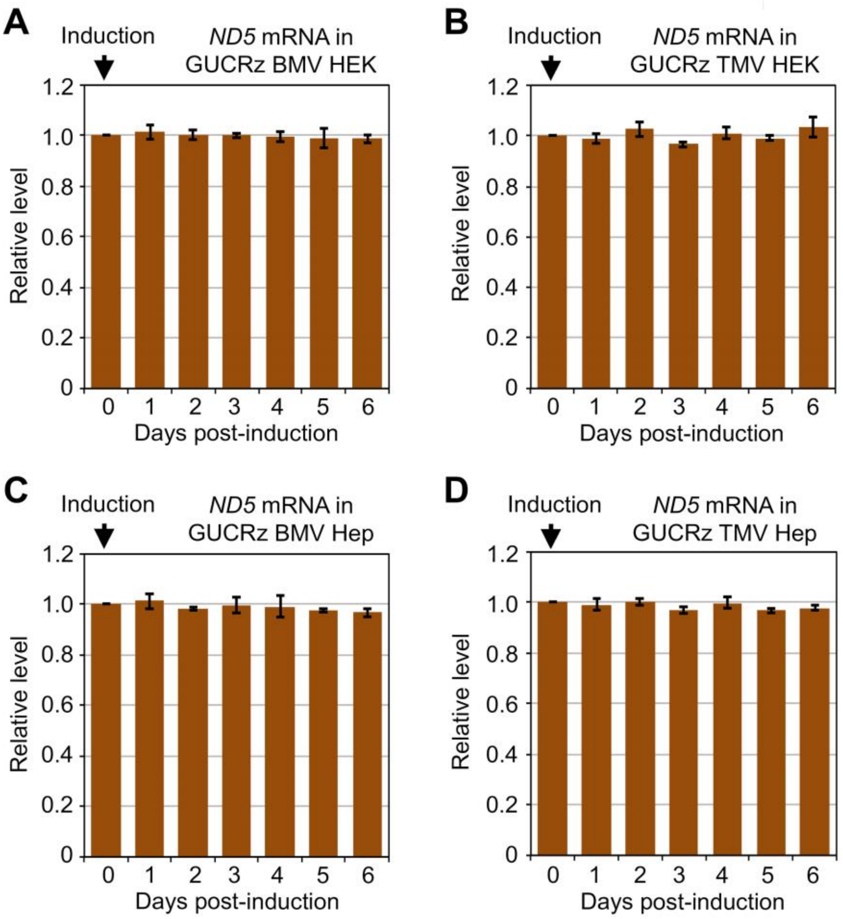

Supplementary Figure S9. Expression of the GUCRz-L-BMV TLS or GUCRz-L-TMV TLS chimeric RNA in HEK (**A** and **B**) or Hep (**C** and **D**) transformants does not affect mitochondrial non-target RNAs. Transgene expression was induced at Day 0 with 10 µg/ml of tetracycline in cells stably transformed with the pcDNA5-cHh-GUCRz-L-BMVTLS-cHDV (**A** and **C**, GUCRz BMV) or pcDNA5-cHh-GUCRz-TMVTLS-cHDV (**B** and **D**, GUCRz TMV) plasmid and samples were subsequently taken every day until Day 6. Total RNA was extracted from all samples and steady-state levels of the mitochondrial *ND5* mRNA were analyzed by RT-qPCR. Data from three independent biological replicates were analyzed. Student's *t*-tests revealed no statistically significant variations. Error bars represent ± SD.

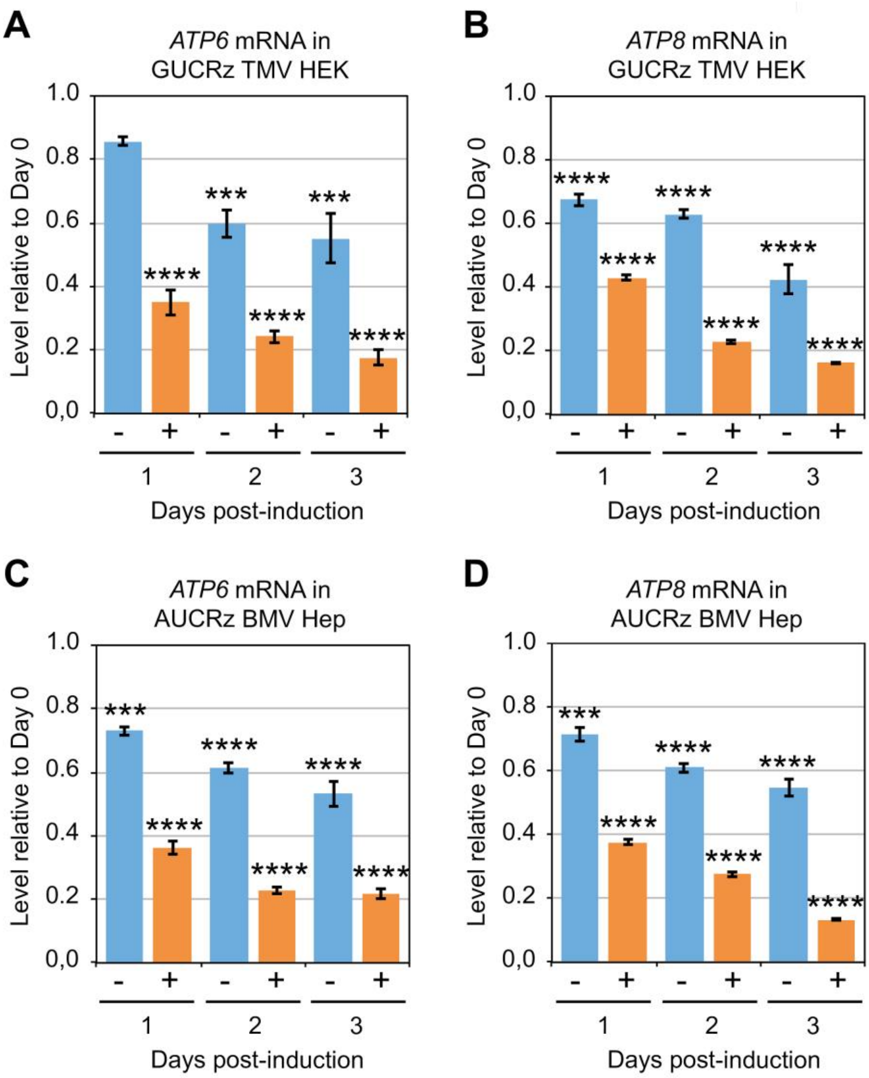

Supplementary Figure S10. Knockdown of the mitochondrial *ATP6* and *ATP8* mRNAs upon expression of the GUCRz-L-TMV TLS or AUCRz-L-BMV TLS chimeric RNA in transformed HEK (**A** and **B**) or Hep (**C** and **D**) cells cultivated under hypoxic conditions. Transgene expression was induced (**+**) or not (**-**) at Day 0 with 10 µg/ml of tetracycline in cells stably transformed with the pcDNA5-cHh-GUCRz-L-TMVTLS-cHDV (**A** and **B**, GUCRz TMV) or pcDNA5-cHh-AUCRz-L-BMVTLS-cHDV (**C** and **D**, AUCRz BMV) plasmid. The cells were subsequently cultivated under hypoxic conditions and samples were taken every day until Day 3. Total RNA was extracted from all samples and steady-state levels of the mitochondrial *ATP6* (**A** and **C**) and *ATP8* (**B** and **D**) mRNAs were analyzed by RT-qPCR. Initial *ATP6* and *ATP8* levels at Day 0 before switching to hypoxic culture conditions were taken as a reference. Hypoxic culture conditions by themselves down regulate the expression of mitochondrial OXPHOS genes (**-**). Target cleavage mediated by the GUCRz or AUCRz ribozyme further knocks down the *ATP6* and *ATP8* mRNAs (**+**). Data from three independent biological replicates were analyzed with the Student's *t*-test; * = $p < 0.05$; ** = $p < 0.01$; *** = $p < 0.001$; **** = $p < 0.0001$. Error bars represent ± SD.

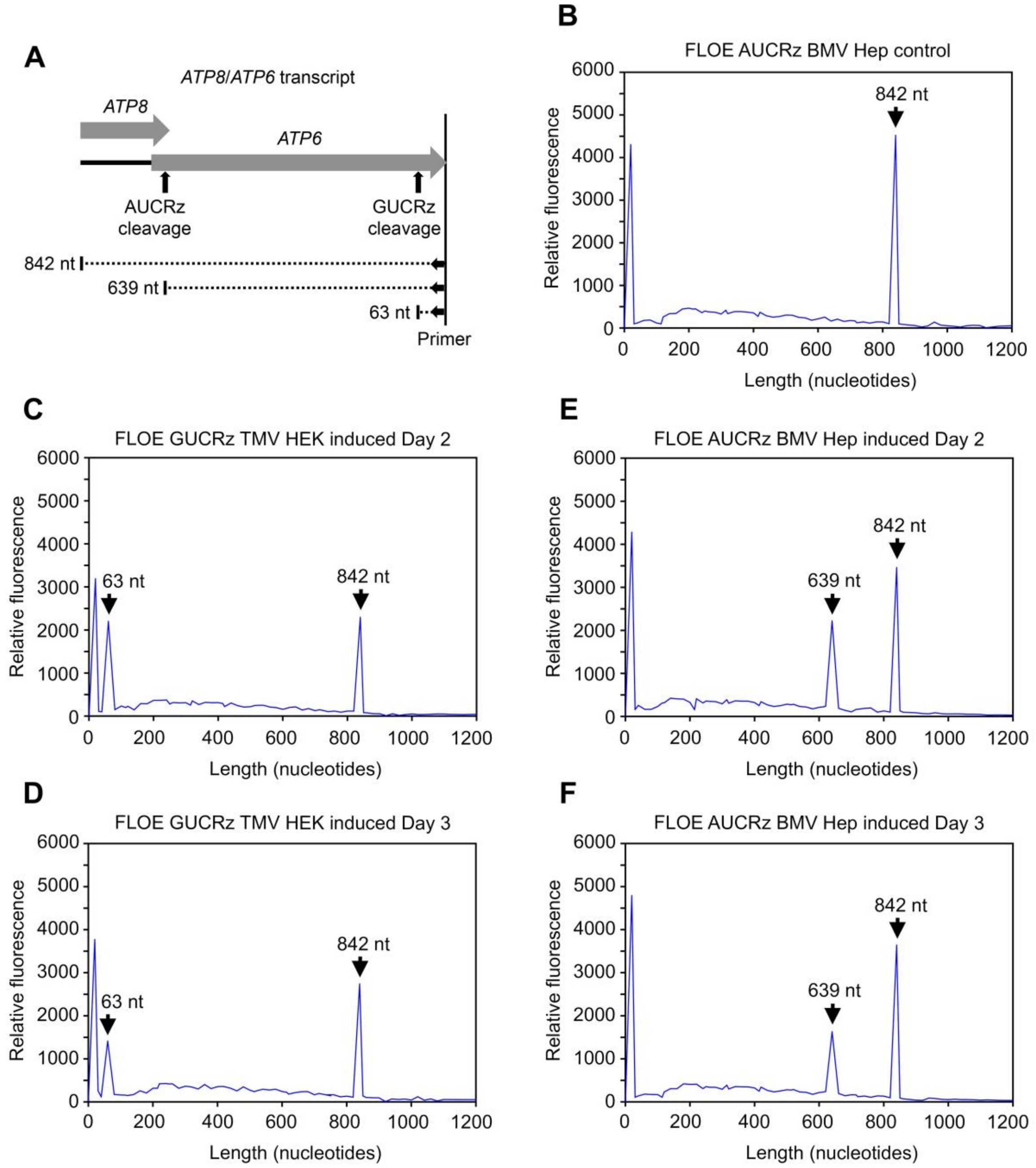

Supplementary Figure S11. *In vivo* cleavage of the mitochondrial *ATP6* mRNA. (**A**) Scheme illustrating the products expected from fluorescently labeled oligonucleotide extension (FLOE) of the full *ATP8*/*ATP6* transcript (842 nucleotides) and of the 3' fragments resulting from cleavage by the AUCRz ribozyme (639 nucleotides) or the GUCRz ribozyme (63 nucleotides), using the atp6_atp8_FAM primer (Supplementary Table S1). (**B**) FLOE analysis of total RNA extracted from control Hep cells, *i.e.* cells stably transformed with the pcDNA5-cHh-AUCRz-L-BMVTLS-cHDV construct but not induced with tetracycline. The 842 nucleotide full length *ATP8*/*ATP6* transcript is indicated by an arrow. (**C** to **F**) Transgene expression was induced at Day 0 with 10 µg/ml of tetracycline in HEK cells stably transformed with the pcDNA5-cHh-GUCRz-L-TMVTLS-cHDV construct (**C** and **D**) or Hep cells transformed with the pcDNA5-cHh-AUCRz-BMVTLS-cHDV construct (**E** and **F**) and samples were subsequently taken every day until Day 3. Total RNA was extracted from all samples and submitted to FLOE analysis. The figure presents the profiles obtained at Day 2 and Day 3. The profiles for Day 1 are shown in Figure 5 of the main part of the manuscript. The FLOE products expected from GUCRz cleavage (63 nucleotides, **C** and **D**) or AUCRz cleavage (639 nucleotides, **E** and **F**) are indicated by arrows. The peak present in all profiles on the extreme left corresponds to the left over of primer.

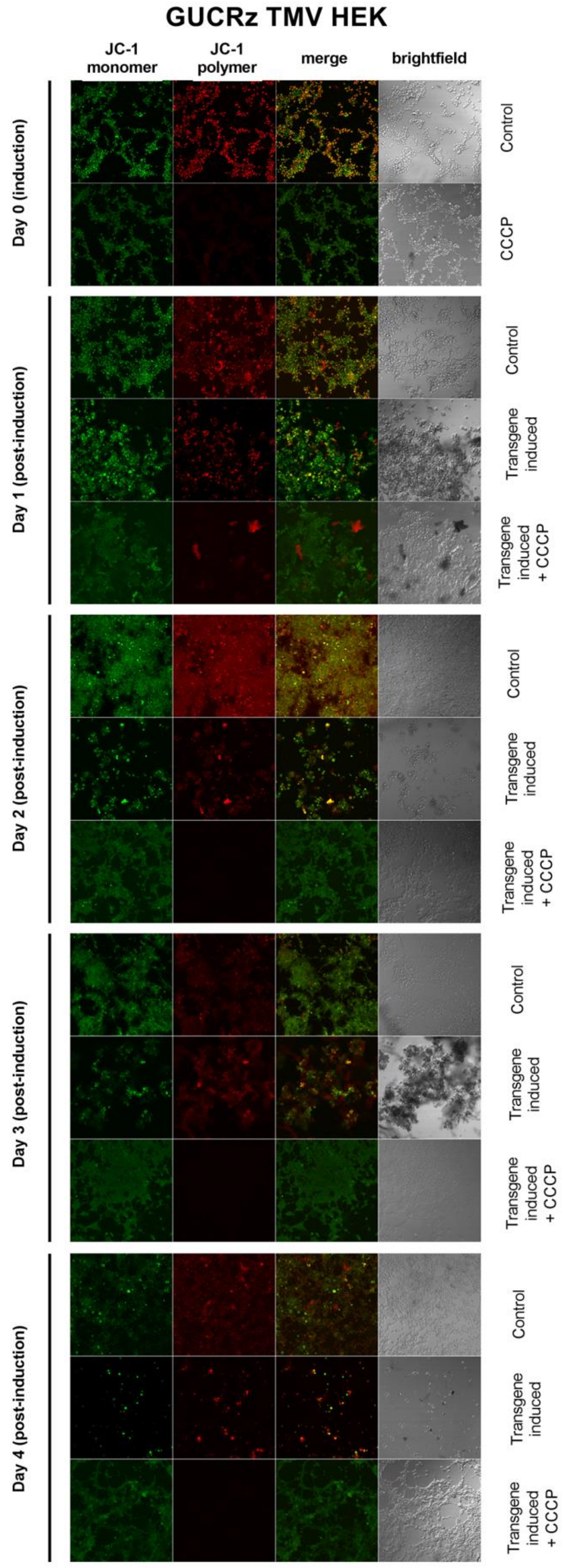

Supplementary Figure S12 Expression of the GUCRz-L-TMV TLS chimeric RNA (GUCRz TMV) was induced at Day 0 with 10 µg/ml of tetracycline in HEK cells stably transformed with the pcDNA5-cHh-GUCRz-L-TMVTLS-cHDV construct. Every day until Day 4, cell samples were incubated for 30 min with JC-1 dye and the fluorescence was measured at 529±5 nm (JC-1 monomer) and 590±5 nm (JC-1 polymer). Addition of the uncoupler CCCP was used as a control of full dissipation of the mitochondrial membrane potential. The panels show representative pictures selected from three independent replicates.

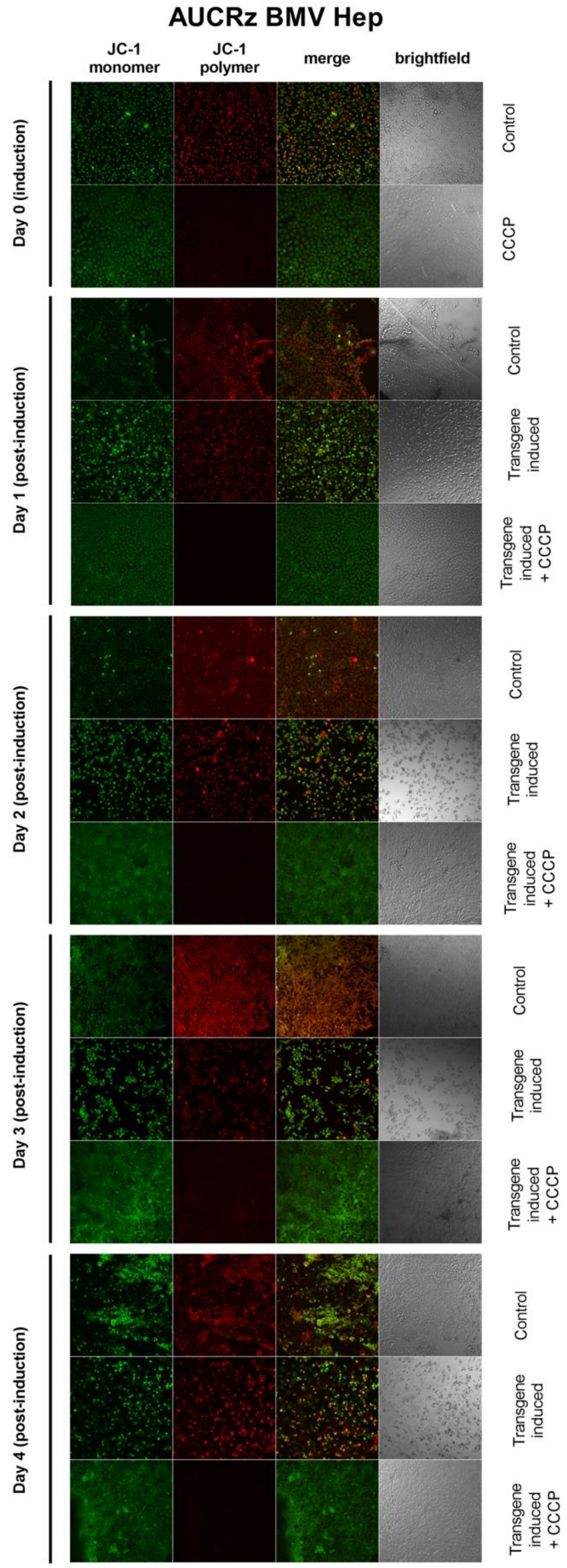

Supplementary Figure S13 Expression of the AUCRz-L-BMV TLS chimeric RNA (AUCRz BMV) was induced at Day 0 with 10 µg/ml of tetracycline in Hep cells stably transformed with the pcDNA5-cHh-AUCRz-L-BMVTLS-cHDV construct. Every day until Day 4, cell samples were incubated for 30 min with JC-1 dye and the fluorescence was measured at 529±5 nm (JC-1 monomer) and 590±5 nm (JC-1 polymer). Addition of the uncoupler CCCP was used as a control of full dissipation of the mitochondrial membrane potential. The panels show representative pictures selected from three independent replicates.

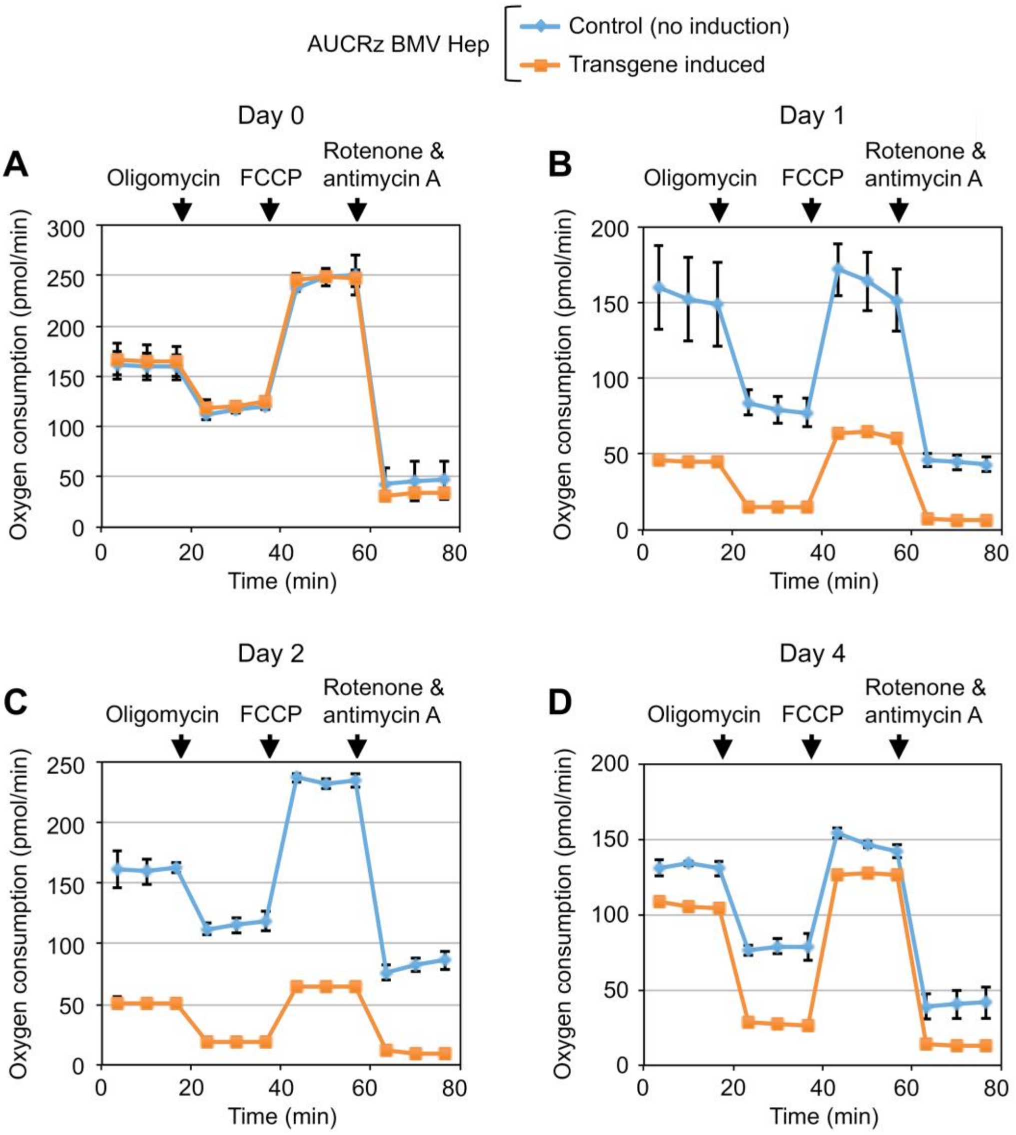

Supplementary Figure S14. Expression of the AUCRz-L-BMV TLS chimeric RNA (AUCRz BMV) in Hep transformants impairs the mitochondrial OXPHOS chain activity. Transgene expression was induced at Day 0 with 10 µg/ml of tetracycline in Hep cells stably transformed with the pcDNA5-cHh-AUCRz-L-BMVTLS-cHDV plasmid and grown in Agilent Seahorse microplates. Control cells were prepared by omitting tetracycline-mediated transgene induction. Oxygen consumption in transgene expressing cells (orange lines) and control cells (blue lines) was subsequently measured every day until Day 4 in an Agilent Seahorse analyzer, with addition of different effectors as indicated (oligomycin, FCCP, rotenone and antimycin A). Data from three independent biological replicates were analyzed. Error bars represent ± SD.

Supplementary Table S1. Primers used in regular PCR, regular RT-PCR, qPCR, RT-qPCR and FLOE reactions. F = forward; R = reverse.

**ACTB_F**	Primers for RT-qPCR analyses	AGAGCTACGAGCTGCCTGAC
**ACTB_R**		AGCACTGTGTTGGCGTACAG
**HPRT F**		TGACCTTGATTTATTTTGCATACC
**HPRT R**		CGAGCAAGACGTTCAGTCCT
**TMV_F**		ACACGTGGTGCGTACGATAA
**TMV_R**		GCTTTATTACGTGCCTGCGG
**BMV_F**		ATAGTCGTGGTTGACACGCA
**BMV_R**		CACTGTACGGTACCCGCATT
**ATP6 F**		GCCGCAGTACTGATCATTCTATT
**ATP6_R**		GGGTGGTGATTAGTCGGTTG
**atp8_F**		ATGGCCCACCATAATTACCC
**atp8_R**		CTAGGATTGTGGGGGCAATG
**Cox1_F**		TCCACGGAAGCAATATGAAA
**Cox1_R**		GGCCACCTACGGTGAAAAG
**Cox2_F**		GATCCCTCCCTTACCATCAAA
**Cox2_R**		GCCGTAGTCGGTGTACTCGT
**Cox3_F**		TGCTTCATCCGCCAACTAAT
**Cox3_R**		AACCACATCTACAAAATGCCAGT
**ND2_F**		GCCCCCATCTCAATCATATACC
**ND2_R**		GTTTAATCCACCTCAACTGCC
**ND5_F**		AAATCCATTGTCGCATCCA
**ND5_R**		TTGGTCTAGGCACATGAATATTGT
**16S_rRNA_1_F**		GTACCGCAAGGGAAAGATGA
**16S_rRNA_1_R**		TTGGCTCTCCTTGCAAAGTT
**pFRTlacZeo_F**	Primers for regular PCR	CGTGACCACATGGTCCTTCT
**pFRTlacZeo_R**		CACTGGCCGTCGTTTTACA
**pcDNA6TR_F**		TGGCGACGCTGTAGTCTTC
**pcDNA6TR_R**		CATTGAAAGAGCAACGGCTA
**H135_F**	Primers for regular RT-PCR	TCCCTATCAGTGATAGAGATCG
**TLSmP_R**		AGAGGGGCGGCAGTTGCACC
**HPro_F**		CGGCTCGTTGGTCTAGGGG
**HPro_R**		GGCTCGTCCGGGATTTGAAC
**HND3_F**		ATAAACTTCGCCTTAATTTTAATAA
**HND3_R**		ATTCGGTTCAGTCTAATCCTTT
**AUCB_1_F**		CCTAGCTGATGAGCTTTTGCG
**AUCB_1_R**		CAACACTGTACGGTACCCGC
**GUCT_1_F**		AAGGCCTGATGAGCTTTTGC
**GUCT_1_R**		GCCCCTACCGGGGGTAA
**ND3_F**		TTTTAATAATCAACACCCTCCTAGC
**ND3_R**		ATTCGGTTCAGTCTAATCCTTTTTG
**AflII-PKTLS5P_F**	PCR primers for assembly of transgene constructs	AGCAACTTAAGACACTTCCACCTAAGTTCTCG
**HDVr-BamHI_R**		GTACAGAATTCTCCCTTAGCCATCCGAGTG
**Hh5PKTLS_F**		AGCAACTTAAGTGTCTGATGAGTCCGTGAGGACG<br>AAACGGTACCCGGTACCGTCACACTTCCA<br>CCTAAGTTCTCG
**BrMVT7_F**		AGCAAGAATTGTAATACGACTCACTATAGGAGCTT<br>GTTGAATCAGTACAATAAC
**BrMVBst_R**		TACAGCCTGGTCTCTTTTAGAGATTTACAGTG
**ToMVT7_F**		AGCAAGAATTGTAATACGACTCACTATAGGGTCAA<br>GATGCATAATAAATAACGG
**ToMVBst_R**		TACAGCCTGGGCCCCTACCGGGGG
**GUCRz_BMV_3Rz_F**		AGCAATGGATCCAGATAATCTGATGAGTCCGTGA<br>GGACGAAACGGTACCCGGTACCGTCATTAAGGCC<br>TGATGAGCTTTTGCGAAACAGCGAGACCAGCAGC<br>TGCCAGTAACACACCTACGTGCGCTGCCAAAGCT<br>TGTTGAATCAGTACAATAAC
**AUCRz_BMV_3Rz_F**		AGCAATGGATCCAGAGCCTCTGATGAGTCCGTGA<br>GGACGAAACGGTACCCGGTACCGTCAGGCCTAG<br>CTGATGAGCTTTTGCGAAATTGTGGGACCAGCAG<br>CTGCCAGTAACACACCTACGTGCGCTGCCAAAGC<br>TTGTTGAATCAGTACAATAAC
**GUCRz_TMV_3Rz_F**		AGCAATGGATCCAGATAATCTGATGAGTCCGTGA<br>GGACGAAACGGTACCCGGTACCGTCATTAAGGCC<br>TGATGAGCTTTTGCGAAACAGCGAGACCAGCAGC<br>TGCCAGTAACACACCTACGTGCGCTGCCAAGTCA<br>AGATGCATAATAAATAACGG
**AUCRz_TMV_3Rz_F**		AGCAATGGATCCAGAGCCTCTGATGAGTCCGTGA<br>GGACGAAACGGTACCCGGTACCGTCAGGCCTAG<br>CTGATGAGCTTTTGCGAAATTGTGGGACCAGCAG<br>CTGCCAGTAACACACCTACGTGCGCTGCCAAGTC<br>AAGATGCATAATAAATAACGG

**HDV_BMV_R**		GTACAGCTCGAGTGGCTCTCCCTTAGCCATCCGAGTGGACGACGTCCTCCTTCGGATGCCCAGGTCGGACCGCGAGGAGGTGGAGATGCCATGCCGACCCTGGTCTCTTTTAGAGATTTACAG
**HDV_TMV_R**		GTACAGCTCGAGTGGCTCTCCCTTAGCCATCCGAGTGGACGACGTCCTCCTTCGGATGCCCAGGTCGGACCGCGAGGAGGTGGAGATGCCATGCCGACCCTGGGCCCCTACCGGGGG
**atp6_atp8_FAM**	Primer for FLOE	***FAM***-TTATGTGTTGTCGTGCAGGTAGAG
**CMV_F**	Primers for sequencing reactions	CGCAAATGGGCGGTAGGCGTG
**BGH_R**		TAGAAGGCACAGTCGAGG
**M13_F**		GTAAAACGACGGCCAG
**M13_R**		CAGGAAACAGCTATGAC

Supplementary References

Bouzaidi-Tiali, N., Aeby, E., Charrière, F., et al. (2007). Elongation factor 1a mediates the specificity of mitochondrial tRNA import in *T. brucei*. EMBO J. *26*, 4302-4312.

Chomczynski, P., and Sacchi, N. (2006). The single-step method of RNA isolation by acid guanidinium thiocyanate-phenol-chloroform extraction: Twenty-something years on. Nat. Protoc. *1*, 581-585.

Dassa, E.P., Dufour, E., Goncalves, S., et al. (2009). Expression of the alternative oxidase complements cytochrome c oxidase deficiency in human cells. EMBO Mol. Med. *1*, 30-36.

Delage, L., Duchêne, A.M., Zaepfel, M., et al. (2003). The anticodon and the D-domain sequences are essential determinants for plant cytosolic tRNA(Val) import into mitochondria. Plant J. *34*, 623-633.

Dietrich, A., Small, I., Cosset, A., et al. (1996) Editing and import: Strategies for providing plant mitochondria with a complete set of functional transfer RNAs. Biochimie *78*, 518-529.

Entelis, N., Brandina, I., Kamenski, P., et al. (2006). A glycolytic enzyme, enolase, is recruited as a cofactor of tRNA targeting toward mitochondria in *Saccharomyces cerevisiae*. Genes Dev. *20*, 1609-1620.

Fechter, P., Rudinger, J., Giege, R., et al. (1998). Ribozyme-processed tRNA transcripts with unfriendly internal promoter for T7 RNA polymerase: Production and activity. FEBS Lett. *436*, 99-103.

Gowher, A., Smirnov, A., Tarassov, I., et al. (2013). Induced tRNA import into human mitochondria: Implication of a host aminoacyl-tRNA-synthetase. PLoS One *8*, e66228.

Huot, J.L., Shikha, S., and Schneider, A. (2019). Inducible orthogonal aminoacylation demonstrates that charging is required for mitochondrial tRNA import in *Trypanosoma brucei*. Sci. Rep. *9*, 10836.

Jeandard, D., Smirnova, A., Tarassov, I., et al. (2019). Import of non-coding RNAs into human mitochondria: A critical review and emerging approaches. Cells *8*, 286.

Jesina, P., Tesarová, M., Fornůsková, D., et al. (2004). Diminished synthesis of subunit a (ATP6) and altered function of ATP synthase and cytochrome c oxidase due to the mtDNA 2 bp microdeletion of TA at positions 9205 and 9206. Biochem. J. *383*, 561-571.

Kore, A.R., Vaish, N.K., Kutzke, U., et al. (1998). Sequence specificity of the hammerhead ribozyme revisited; the NHH rule. Nucleic Acids Res. *26*, 4116-4120.

Laforest, M.J., Delage, L., and Maréchal-Drouard L. (2005). The T-domain of cytosolic tRNAVal, an essential determinant for mitochondrial import. FEBS Lett. 579, 1072-1078.

Lloyd, A.L., Marshall, B.J., and Mee, B.J. (2005). Identifying cloned *Helicobacter pylori* promoters by primer extension using a FAM-labelled primer and GeneScan analysis. J. Microbiol. Methods *60*, 291-298.

Ludwig, J., Blaschke, M., and Sproat, B.S. (1998). Extending the cleavage rules for the hammerhead ribozyme: Mutating adenosine15.1 to inosine15.1 changes the cleavage site specificity from N16.2U16.1H17 to N16.2C16.1H17. Nucleic Acids Res. *26*, 2279-2285.

Perales-Clemente, E., Fernandez-Silva, P., Acin-Perez, R., et al. (2010). Allotopic expression of mitochondrial-encoded genes in mammals: Achieved goal, undemonstrated mechanism or impossible task ? Nucleic Acids Res. *39*, 225-234.

Perrotta, A.T., and Been, M.D. (2007). A single nucleotide linked to a switch in metal ion reactivity preference in the HDV ribozymes. Biochemistry *46*, 5124-5130.

Quirós, P.M., Mottis, A., and Auwerx, J. (2016) Mitonuclear communication in homeostasis and stress. Nat. Rev. Mol. Cell Biol. *17*, 213-226.

Schneider, A. (2011). Mitochondrial tRNA import and its consequences for mitochondrial translation. Annu. Rev. Biochem. *80*, 1033-1053.

Seo, B.B., Nakamaru-Ogiso, E., Flotte, T.R., et al. (2006). *In vivo* complementation of complex I by the yeast Ndi1 enzyme. Possible application for treatment of Parkinson disease. J. Biol. Chem. *281*, 14250-14255.

Sharma, A., and Sharma, A. (2015). *Plasmodium falciparum* mitochondria import tRNAs along with an active phenylalanyl-tRNA synthetase. Biochem J. *465*, 459-469.

Silva-Pinheiro, P., Nash, P.A., Van Haute, L., et al. (2022). *In vivo* mitochondrial base editing via adeno-associated viral delivery to mouse post-mitotic tissue. Nat. Commun. *13*, 750.
